# Projected slow down of South Indian Ocean circulation

**DOI:** 10.1038/s41598-019-54092-3

**Published:** 2019-11-27

**Authors:** Annette Stellema, Alex Sen Gupta, Andréa S. Taschetto

**Affiliations:** Climate Change Research Centre and Australian Research Council Centre of Excellence for Climate Extremes, University of New South Wales, Sydney, New South Wales 2052 Australia

**Keywords:** Physical oceanography, Physical oceanography

## Abstract

Using an ensemble of 28 climate models, we examine hindcasts and ‘business as usual’ future changes to large-scale South Indian Ocean dynamics. We compare model ensemble seasonal-to-annual volume transports to observations and explore drivers of past and future circulation variability and change. Off the west coast of Australia, models consistently project a weakening of the Leeuwin Current and Undercurrent due to reduced onshore flow and downwelling. The reduced onshore flow is related to changes in the alongshore pressure gradient. While the alongshore pressure gradient change is consistent with the Indonesian Throughflow projected weakening, we found no inter-model relationship between these changes. In the south-western Indian Ocean, the models project a robust weakening of the North East and South East Madagascar Currents, Agulhas Current and transport through the Mozambique Channel. This reduced Indian Ocean western boundary flow is partly associated with a weaker Indonesian Throughflow and overturning circulation, where the latter is related to a decrease in the convergence of deep Southern Ocean waters into the Indian Ocean. In contrast to the weakening of other features, the westward flowing Agulhas Current extension south of Africa is projected to strengthen, which is consistent with an intensification of the Antarctic Circumpolar Current.

## Introduction

The circulation of the South Indian Ocean modulates marine life and global climate through important oceanic teleconnections with the Pacific, Atlantic and Southern Oceans. Its circulation is distinctive to the other tropical oceans in numerous ways. Unlike the Pacific and Atlantic that have sustained equatorial and eastern boundary upwelling, in the Indian Ocean the shallow upwelling of cool, nutrient-rich waters primarily occurs north of the equator and east of Madagascar^1^. There is also a unique tropical conduit between the Pacific and the Indian Ocean, the Indonesian Throughflow (ITF), which helps set up a large meridional pressure gradient in the south-eastern basin. This meridional pressure gradient drives flow towards the west coast of Australia, which feeds the southward flowing Leeuwin Current (LC)^2^. In the south-western basin, the southward flowing South East Madagascar Current (SEMC) and transport through the Mozambique Channel (MZC) help supply the energetic Agulhas Current (AC)^3–5^. Through a complex interplay of nonlinear, mesoscale processes to the south of Africa, warm and saline Indian Ocean waters leak into the Atlantic and contributes to the Atlantic Meridional Overturning Circulation^3,6^.

A limited number of studies examined future changes to the Indian Ocean circulation and suggest substantial changes are likely. For example, a study using an eddy-permitting ocean model forced by projection information from a CMIP3 model found that the LC will decrease in the 21^st^ century^7^. The study postulated that this was related to a projected decrease in sea level pressure and ITF transport^7^. Future variations of the LC can have consequences for regional weather and the marine ecosystem off the west coast of Australia. The latter is still in recovery after decades of ocean warming and an unprecedented marine heatwave in 2011^8^.

Observations indicate a multi-decadal warming trend of the South Indian Ocean gyre^9^ and AC region^10^, likely as a result of a southward shift and intensification of the southern hemisphere westerlies^9,10^. The intensifying winds are also thought to have increased western boundary eddy activity, causing the observed broadening of the AC since the 1990s^11^, although the relationship between southern wind changes and trends in AC transport has been questioned^12^. In addition, observational and modelling results indicate that Agulhas leakage into the Atlantic has increased in response to these wind changes over the last few decades^13^. Projections based on an eddy-permitting model forced by an atmosphere model subject to doubled CO_2_ concentrations projected a decrease of AC transport and an increase of Agulhas leakage^14^. To date, there have been no studies that examine projected changes of the currents east of Madagascar or the transport through the MZC. It is important to understand how the circulation along the east African coast is likely to change as it regulates regional climate conditions and ecosystems, which have considerable importance for Africa’s economic development^15^.

Here, we provide the first multi-model (MM) examination of the long-term projected changes of South Indian Ocean circulation in a future of increasing greenhouse gas emissions. We use an ensemble of 28 climate models taking part in the Coupled Model Intercomparison Project Phase 5 (CMIP5) to compare MM ensemble volume transports to observations and provide an estimate of structural uncertainty in projections. We focus on large-scale circulation features, on long-term mean seasonal-to-annual timescales and examine mechanisms that may be driving projected changes. In particular, we examine the LC system consisting of the LC, Leeuwin Undercurrent (LUC) and the interconnecting zonal and vertical flows. To help understand projected LC system changes, we investigate the influence of changes in the local meridional wind stress and alongshore pressure gradient. In the south-western Indian Ocean, we examine the North East Madagascar Current (NEMC), SEMC, AC and transport through the MZC. To help understand the mechanisms driving projected changes of these features, we investigate the influence of changes in interior transport, basin-wide wind forcing, the ITF and links to the Southern Ocean.

## Methods

### Climate models

We used output from 28 CMIP5 climate models (listed in Supplementary Table S1) and examined two experiments: (i) the historical scenario forced with observationally derived data of natural and anthropogenic forcing, and (ii) the RCP8.5 scenario, which assumes a rapid increase of greenhouse gases over the 21st century that reaches ~1370 ppm CO_2_-equivalent concentrations in 2100^16^. To examine long-term projected changes, we compare simulation averages over two periods: 1900–2000 from the historical scenario and 2050–2100 from RCP8.5. The multi-decadal timescales allow sufficient time for the ocean to adjust to surface forcing changes and minimise noise associated with low-frequency internal variability. Due to the seasonal nature of many South Indian Ocean currents, we examined the long-term monthly and annual climatologies for both periods.

We used a single ensemble member from each model (usually r1i1p1) that archived the ocean and atmospheric variables: seawater velocity and wind stress. We used a subset of 22 out of 28 models for sea surface height calculations, as that variable was unavailable in the accessed repository at the time that we conducted the study for models: CESM1-CAM5-1-FV2, CESM1-CAM5, GFDL-CM3, HadGEM2-AO, MIROC5 and NorESM1-ME. We did not correct for model drift because any drift in the circulation is likely to be less significant than forced future changes and is presumed to be negligible in the MM mean^17^.

### Transport and Sverdrup dynamics

We calculated transport by integrating seawater velocity on each model’s native grid, where the integration boundaries (e.g. width and depth) are manually selected based on an examination of each model’s long-term annual mean velocity profile. The boundary currents generally follow the coastlines, so we integrated the velocity between the coast and a vertically uniform distance offshore. Because the offshore extent of the various currents varies considerably between models, we subjectively selected a boundary to suit each model based on an examination of the historical mean meridional flow at different latitudes along the extent of the current. In particular, we defined a distance offshore (given as X degrees offshore) for each model that remained constant with latitude and depth. The offshore distance was chosen, as far as possible, to include the current in question and minimise the inclusion of any counter current. In a few cases, we needed to manually adjust the distance offshore at selected latitudes when the current clearly moved beyond the boundary. Due to the complex structure of the weak LC and LUC, a common offshore distance (~5° offshore) was used that encompassed these currents in all models. Our static integration boundaries may neglect offshore seasonal meandering and projected changes that are related to structural changes such as broadening or deepening. However, we performed sensitivity analyses and found the transport values and projections are not particularly sensitive to our integration boundaries.

For most circulation features, we integrated the net velocity to calculate transport. We only integrated velocity in the direction of the mean flow for the LC, LUC and AC extension transports, as their integration boundaries included a considerable amount of counter-flowing areas. Although, we used net LC and LUC transport in the budget calculations to ensure a closed budget. To validate our results, we compared MMM transports to observational estimates, the latter we collated in Supplementary Table S2.

Projected changes to South Indian Ocean upper-interior transport were compared to expected wind-driven changes using Sverdrup dynamics. The Sverdrup model of depth-integrated flow states that the meridional advection of planetary vorticity is balanced by the wind stress curl, in the absence of deep density-driven circulation. Sverdrup theory is applicable for this study because we consider the quasi-steady-state conditions of multi-decadal timescales. As there are considerable inter-model differences in resolution and representations of coastlines, to calculate the Sverdrup transport we linearly interpolated each model’s wind stress to a common 0.5° x 0.5° grid. Sverdrup transports from FIO-ESM, MIROC-ESM and MIROC-ESM-CHEM models should be treated with caution as their wind stress curl product shows some unrealistic banding. However, their Sverdrup transports lie within the range of other models and their inclusion has little impact on our overall results (see Supplementary Table S3 for individual model Sverdrup transports).

### Statistical analysis

We typically provide multi-model median (MMM) metrics and the associated interquartile range (IQR), as the median is less affected by outlier models than the arithmetic mean. However, we give the MM mean for transport budgets because medians would not provide closed budgets. We listed all long-term MMM, MM mean and individual model transports in Supplementary Table S3. To determine the significance level of projected changes, we applied the non-parametric Wilcoxon signed-rank test^18^. We considered changes to be statistically significant if they are above the 95% level (p < 0.05). We measured the degree of a monotonic relation between two variables by applying the non-parametric Spearman rank correlation^19^ (r_s_), where the associated significance is based on a 2-tailed p-value test. There are caveats concerning the validity of these statistical tests as the assumption of sample independence may not be satisfied (e.g. models are not independent of each other and share common components; see Supplementary Table S1) and our ensemble of models does not represent all possible variations of the climate system.

## Results

### Indonesian Throughflow

**Figure 1:**
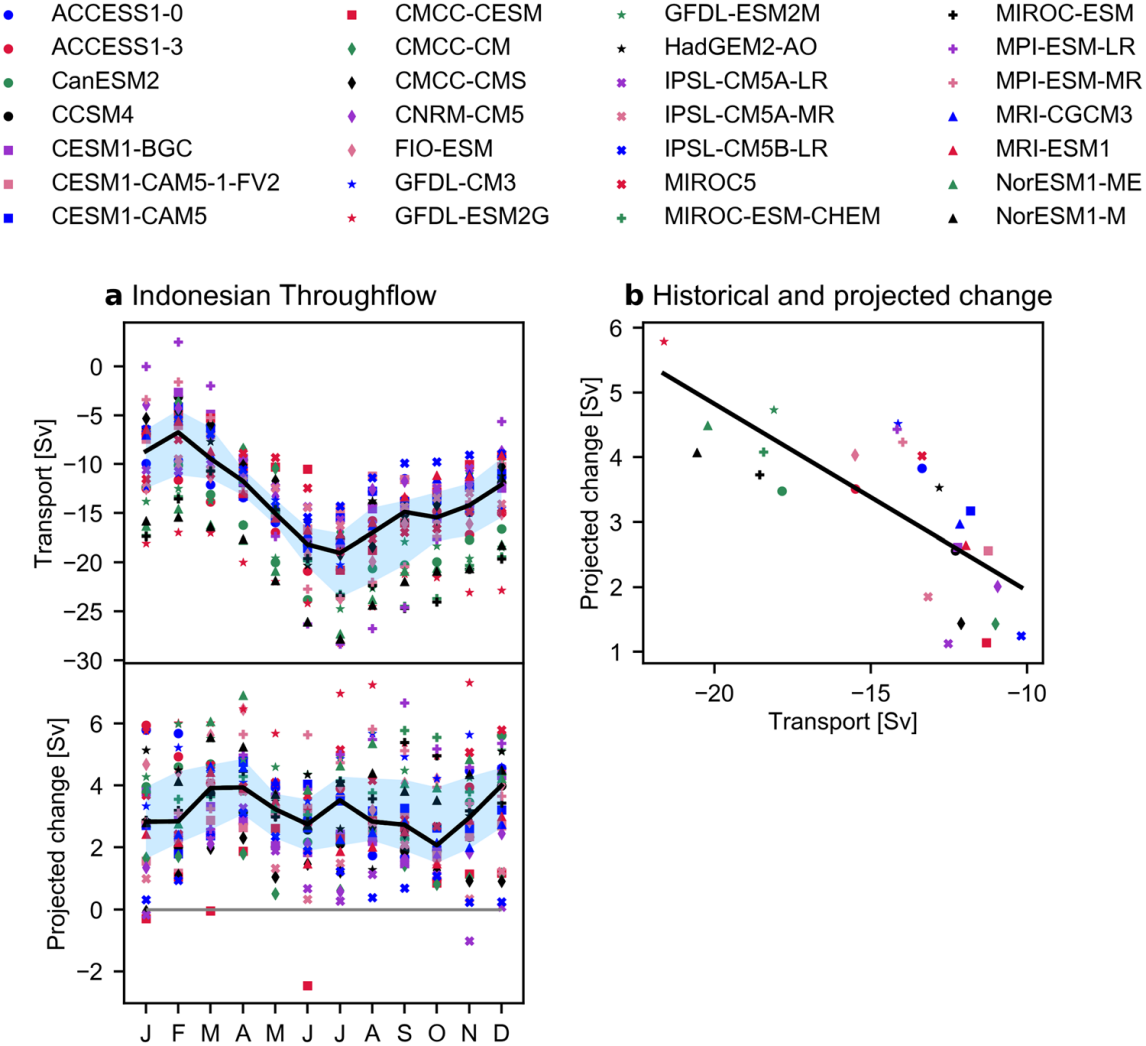
Indonesian Throughflow historical and projected transport change. (**a**) Monthly transport (Sv) in the historical scenario (top panel) and projected change (RCP8.5 minus historical; bottom panel). (**b**) Relationship between long-term mean historical transport and associated projected change. Westward transport is represented by negative values, where a corresponding positive change indicates a decrease in transport. Coloured markers indicate individual model transport estimates (see legend at the top). In (**a**), the multi-model median (solid lines) and interquartile range (shaded blue) are shown. The multi-model median projected change is statistically significant at the 95% level during all months.

We calculated the ITF transport into the Indian Ocean as the net zonal transport shallower than 1500 m and between the most western point of Australia and Indonesia. Our definition of the ITF may neglect a small proportion of the total transport, through straits between Indonesian Islands lying to the west of this point, in some models. In the historical scenario, the MMM ITF is −13 Sv (1 Sv ≡ 10^6^ m^3^ s^−1^; negative values indicate westward flow) with a relatively small IQR of −12 to −16 Sv. The MMM transport is slightly lower than the long-term current meter mooring estimate of −15 Sv^20^, although observational estimates remain uncertain (see range of estimates in Supplementary Table S2). The simulated ITF transport has a distinct seasonality (Fig. 1a; top panel): the MMM transport is largest in July (−19 Sv) and lowest in February (−7 Sv). The MMM seasonality is in remarkable agreement with observations, which is mainly associated with local monsoon wind forcing^20^.

The ITF transport is projected to decrease by a MMM of 3.5 Sv (IQR 2.4 to 4.1 Sv), corresponding to a substantial 26% transport decrease. Generally, there is no clear model agreement on the seasonality of the projected change (Fig. 1a; bottom panel). For example, the largest MMM weakening occurs in December, yet only 4 out of 28 models agree this is the month of maximum change (Fig. 1a). As shown in Fig. 1b, models that simulate larger mean ITF transport also tend to project a larger weakening (r_s_ = −0.79, p < 0.001). This robust relationship can be used to provide an emergent constraint on the future ITF transport change: based on an observed transport of −15 Sv^20^ we would expect a change of 3 to 4 Sv (Fig. 1b), which is similar to the MMM of 3.5 Sv.

### Leeuwin Current system

#### Leeuwin Current (LC)

All models simulate an LC-like surface intensified southward flow along the west coast of Australia between 22°S and 33°S. We defined LC transport, based on the annual mean velocity, as the southward component of flow above 200 m and within ~5° from the coast. There is considerable inter-model spread in the structure (e.g. depth and width) of the LC that varies with latitude and season. Consequently, this definition in some models may exclude offshore seasonal meandering and include a component of neighbouring southward flow, where the latter is typically prominent towards the north.

**Figure 2:**
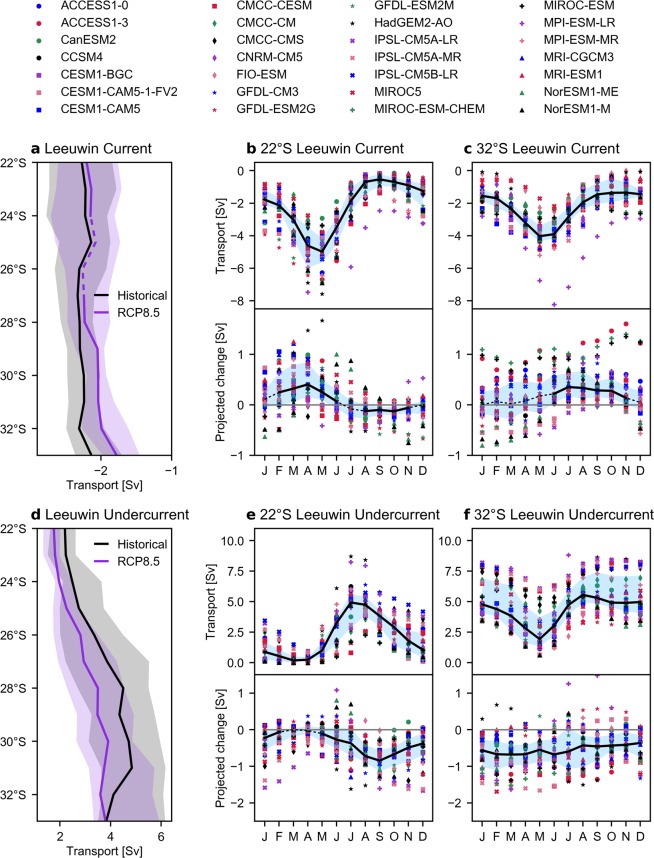
Leeuwin Current and Leeuwin Undercurrent historical and projected transport change. (**a**) Leeuwin Current long-term multi-model median transport (Sv) in the historical (black) and RCP8.5 (purple) scenario, where the associated interquartile range is shaded. (b) Leeuwin Current monthly transport at 22°S in the historical scenario (top panel) and projected change (bottom panel). (**c**) Same as (**b**), but at 32°S. (**d**), Same as (**a**), but for the Leeuwin Undercurrent. (**e**) Same as (**b**), but for the Leeuwin Undercurrent. (**f**) Same as (**e**), but at 32°S. Shown in (**b,c**) and (**e,f**) are the multi-model median (black line), interquartile range (shaded blue), individual model (markers; see the legend at the top) transport estimates and, in the bottom panels, a grey zero-line for reference. Negative values represent southward transport and a corresponding positive change indicates a decrease in transport. Dashed lines represent projected changes that are not statistically significant at the 95% level.

In the historical scenario, the MMM LC transport is −2.3 Sv (−1.8 to −2.7 Sv) at 22°S and similarly −2.3 Sv (−2.0 to −2.5 Sv) at 32°S (negative values indicate southward transport; Fig. 2a). The MMM LC transport is within the range of observational estimates (e.g. −1.5 Sv at 34°S^21^ to −3.4 Sv^22^ at 32°S), although estimates vary considerably (Supplementary Table S2). We do not find a southward intensification of the MMM LC (Fig. 2a). This is consistent with results from gridded hydrographic data^21^. However, this lack of southward intensification is inconsistent with the theory that the LC intensifies as it is continually fed by eastward Indian Ocean waters^23–25^. The inconsistency may be related to the water loss through downwelling along the LC path (see the Transport budget section). The simulated LC has a strong seasonality that is consistent across most models and is in broad agreement with observations^21,26,27^. Specifically, LC transport peaks in May at all latitudes, with maximum transports of −5.0 Sv and −4.0 Sv at 22°S and 32°S, respectively (Fig. 2b,c; top panels).

There is a projected decrease in LC strength along the west coast of Australia (Fig. 2a). The magnitude of MMM changes are relatively low, with considerable model spread and the change is not statistically significant at 25°S and 26°S (Fig. 2a). The largest projected change of annual mean transport is 0.23 Sv (−0.01 to 0.35 Sv) at 33°S corresponding to a decrease of 11%. The LC projected decrease shows a distinct latitude-dependent seasonality. At 22°S, the largest change of 0.4 Sv occurs around February–March (Fig. 2b; bottom panel), while further south at 32°S the largest change of 0.35 Sv occurs around July–August (Fig. 2c; bottom panel). The projected change is not significant for all months (Fig. 2b,c; dashed lines). At 22°S, the MMM LC transport is projected to increase around July–November, which is when the LC transport is typically the weakest (Fig. 2b). Unlike the ITF transport, there is no significant relationship between LC transport and the projected change at any latitude (not shown).

#### Leeuwin Undercurrent (LUC)

The LUC is calculated as the northward component of transport between 200 m and 800 m, using the same offshore boundaries as the LC. The offshore boundary may underestimate the full transport in some of the models that simulate an unrealistically broad undercurrent. The annual mean LUC transport shows an equatorward weakening (Fig. 2d), which is generally consistent with results from previous observational^21^ and modelling^28^ studies. Although, a part of this change may be related to our definition because the LUC tends to shoal upward and move offshore as it flows equatorward in most models. At 22°S, LUC transports a MMM of 2.2 Sv (1.6–2.6 Sv) northward which increases to a maximum of 4.8 Sv (3.6–6.2 Sv) at 31°S (Fig. 2d), which is within the range of hydrographic estimates (1.7–5 Sv^21,29^; see Supplementary Table S2). Generally, LUC transport peaks around July–August (Fig. 2e,f; top panels) along the Western Australia coast in the models. The minimum monthly transport occurs around March–May, with the northward flow almost disappearing in these months towards the north (Fig. 2e,f). Based on the net transport, the LUC reverses direction during these months in many models. The seasonality is generally consistent with hydrographic estimates that show the monthly transport maximum and minimum occurs in October and May, respectively^21^. There are considerable inter-model differences in monthly LUC transport, as shown in Fig. 2e,f, where the LUC transport in some models (e.g. CMCC-CESM, IPSL-CM5A-LR and IPSL-CM5B-MR) is much larger than the MMM. Based on inspection of the annual velocity, the simulated LUC core in these models is much larger and closer to the coast compared to the other models. In several models, the simulated LUC meanders offshore during March–May, particularly towards the north, which will appear as a decrease in transport in our calculations. This meandering may also contribute to the observed annual equatorward weakening (Fig. 2d).

In the last half of the 21^st^ century, the annual mean LUC transport is projected to weaken significantly at all latitudes (Fig. 2d). The highest magnitude projected change is at 28°S, where all models project a weakening, with MMM of −0.67 Sv (−0.42 to −1.1 Sv), corresponding to a 15% decrease from 4.5 Sv (2.7 to 5.5 Sv). There appears to be a seasonality in the projected change towards the north that is consistent across most models. Specifically, at 22°S, there is a significant weakening in May–February that is largest around September (Fig. 2e; bottom panel). Further south, there is no clear model agreement on the projected weakening seasonality (Fig. 2f; bottom panel). In contrast to the LC, the LUC historical transport and projected change is highly correlated at most latitudes, such that models with the largest mean transport generally show the greatest weakening (r_s_ = −0.76, p < 0.001 at 22°S; r_s_ = −0.40, p = 0.03 at 32°S).

#### Transport budget

**Figure 3:**
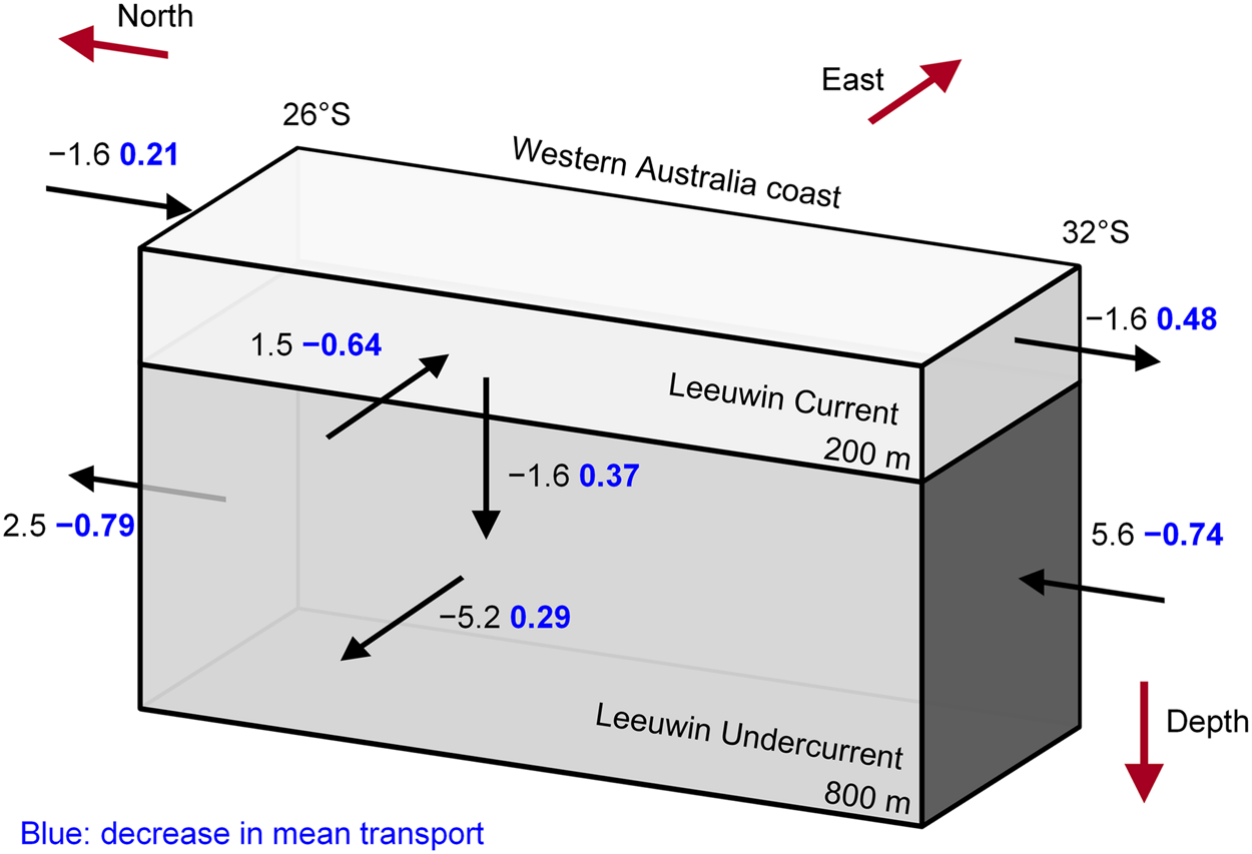
Leeuwin Current system transport budget. Transport budget of the Leeuwin Current system between 26°S and 32°S including the multi-model mean net transport (Sv) in the historical scenario (black) and the projected change (blue if weakening). Eastward, northward and upward transport is defined as positive, where arrows indicate mean transport direction, but not magnitude. All projected changes shown are statistically significant at the 95% level. The vertical transport below the Leeuwin Undercurrent is not shown. See text for boundary definitions.

To examine changes to the complete LC system and how the meridional transport links to zonal and vertical transport changes, we perform a transport budget analysis. We define a transport box containing the LC system where we calculate the *net* transport across the northern, southern and offshore faces and calculate downwelling from continuity (Fig. 3). We calculate the budget between 26°S and 32°S, where most models simulate strong onshore transport into the LC. For the offshore face, we integrate the zonal flow along a fixed transect that is ~5° from the coast at 26°S to 32°S. To close the transport box, the offshore boundary at 32°S may be modified (usually increased) because the coastline varies between the latitudes. The depth definition of the LC and LUC remains as 0–200 m and 200–800 m, respectively (Fig. 3). A small error may be introduced as we assume the transport through the bottom of the LUC box is negligible (MM mean of −0.5 Sv). We calculate budgets for individual models before calculating MM means. Note that as we are necessarily using net MM mean values (see Methods), our transports will differ slightly from the previous sections that presented unidirectional median transports.

In the budget, the mean LC transport does not change much with latitude (both −1.6 Sv), while the LUC weakens considerably as it flows northward (5.6 Sv to 2.5 Sv; Fig. 3). The associated zonal circulation is asymmetric, that is, less water flows onshore (1.5 Sv) than the subsurface offshore flow (−5.2 Sv) since the onshore transport almost equals the downwelled transport (−1.6 Sv; Fig. 3). The MM mean onshore and vertical flows are lower than observed hydrographic estimates between 22°S and 34°S, of −4.7 Sv and −3.4 Sv, respectively^21^. Nearly all models simulate westward flow out of the LUC between the north and south boundaries, which is in disagreement with gridded hydrography estimates that found eastward flow south of 28°S feeds into the LUC^21^, but in agreement with previous numerical model simulations^30^. It was suggested that this model-observation discrepancy results from inadequate simulation of the Flinders Current retroflection in this region^21^.

In the future, all branches of LC system transport are projected to weaken significantly, as shown in Fig. 3. The LC transport weakens more at the southern boundary (0.48 Sv; −29%) than it does further north (0.21 Sv; −13%) because the substantial weakening of onshore flow (−0.64 Sv; −41%) is greater than the reduction in downwelling (0.37 Sv; −24%). The weakening of downwelling and offshore flow at depth (0.29 Sv; −5%) is similar and as such the LUC weakens at the northern and southern boundaries by a similar amount (Fig. 3).

#### Drivers of change

The alongshore momentum balance for the LC is a balance between the meridional winds, the meridional pressure gradient and bottom stress^26^. LC system variability is thought to be linked to changes in the prevailing southerly winds on seasonal timescales^23,26,27,31^ and by the *El Niño*–Southern Oscillation events driving changes to the ITF, and subsequently the meridional pressure gradient along the west coast of Australia on inter-annual timescales^27,32,33^. In this section, we examine processes that may help explain inter-model differences in the mean historical state and the projected change. In particular, we consider three relationships: (i) the effect of local meridional wind stress on the strength of the LC and vertical transport, (ii) the effect of the meridional pressure gradient on the LC system strength, and (iii) the influence of ITF strength on the meridional pressure gradient. To test these relationships, we use the annual mean unidirectional meridional transport and the zonal and vertical transport as calculated in the transport budget. In addition to examining inter-model differences, we examine the inter-annual variability of the meridional pressure gradient in three models that simulate moderate, low and high ITF/ LC transport, respectively: ACCESS1-0, GFDL-ESM2M and MRI-ESM1. We examine LC system transport from 1901 to 2000, where correlations are based on annual mean anomalies for each year.

To help understand the importance of regional meridional winds on LC system transports, we consider the meridional wind stress averaged over the surface of the LC system box (Fig. 3), giving a MMM of 7.2 × 10^−2^ N m^−2^ (5.9–7.8 × 10^−2^ N m^−2^; southerly), broadly consistent with the 6.0 × 10^−2^ N m^−2^ observed estimate^27^. Based on historical transports, we do not find significant inter-model correlations between the upwelling-favourable southerlies and LC strength or downwelling on interannual timescales (Table 1). These findings are unsurprising as studies find that the annual mean wind-induced Ekman drift is negligible compared to geostrophic transports for most months of the year^26,27^. All but one model project a strengthening of the southerlies, with a statistically significant MMM increase of 0.55 × 10^−2^ N m^−2^ or 8% (0.31–0.96 × 10^−2^ N m^−2^). The wind stress strengthening is consistent with increased offshore Ekman transport, reduced downwelling, and the subsequent slowing of the LC system^34^. Despite the consistent MMM change, we find no significant inter-model relationship between the southerlies and the vertical or LC transport projected changes (Table 1) – i.e. differences in projected wind strength across the models cannot explain inter-model differences in change in the LC transport.

**Table 1:** Correlation coefficients between annual mean Leeuwin Current system transport and the meridional wind stress or sea surface height gradient (ΔSSH).

	Meridional wind stress		ΔSSH				
	H	Δ	H	Δ	Inter-annual		
					ACCESS1-0	GFDL-ESM2M	MPI-ESM1
26°S ${V}_{{LC}}$	−0.37	−0.15	0.26	−0.19	−0.09	−**0.35**	−0.15
26°S ${V}_{{LUC}}$	—	—	**0.46**	−0.09	**0.21**	**0.50**	**0.21**
32°S ${V}_{{LC}}$	0.02	−0.21	−0.35	**−0.53**	−0.11	−**0.38**	−0.19
32°S ${V}_{{LUC}}$	—	—	**0.43**	0.36	**0.33**	**0.47**	**0.35**
${U}_{{LC}}$	—	—	**0.84**	**0.81**	**0.48**	**0.82**	**0.76**
${U}_{{LUC}}$	—	—	−0.25	**−0.59**	**−0.22**	**−0.72**	**−0.41**
${W}_{{LC}}$	0.08	0.11	**−0.77**	−0.06	**−0.49**	**−0.34**	−0.01
ITF	—	—	−0.16	−0.07	−0.13	**−0.48**	**−0.47**

H: historical. Δ: RCP8.5 projected change. Leeuwin Current system transports including meridional (V), zonal (U), and vertical (W) transport. LC: Leeuwin Current. LUC: Leeuwin Undercurrent. ITF: Indonesian Throughflow. Bold coefficients indicate a significant correlation at the 95% confidence level. The inter-annual columns include the correlation coefficient for models ACCESS1-0, GFDL-ESM2M and MPI-ESM1.

The meridional pressure gradient, which is associated with the shallow onshore/deep offshore geostrophic flow, is calculated as the difference in sea surface height (ΔSSH) averaged over 20–25°S and 30–35°S between 108–113°E (north minus south section). As expected, we find that on inter-annual timescales, a larger ΔSSH corresponds to enhanced shallow onshore and deep offshore flow in most cases (Table 1). Additionally, the larger ΔSSH is associated with enhanced LC transport in one of the three models and diminished LUC transport in all three models (Table 1). We also find a significant relationship between ΔSSH and ITF transport on inter-annual timescales in two of the three models tested (Table 1). The lack of a significant correlation between ΔSSH and ITF transport in ACCESS1-0 suggests that the Kelvin waves, generated by inter-annual variations winds over the western Pacific, are not reaching the coast off Western Australia as they do in observations^35^. Nevertheless, ITF transport will affect the large-scale meridional pressure gradient that drives the onshore geostrophic flow that feeds the LC on longer timescales.

The long-term MMM ΔSSH is 0.29 m (0.25–0.31 m), consistent with the positive observed sea level difference of about 0.33–0.5 m^27,34,36^. Interestingly, the MMM ΔSSH is not projected to change significantly under RCP8.5. We note that there is a statistically significant weakening of ΔSSH from August to September, where 15/22 models agree on a decrease in August (similar timing to LC weakening at 32°S; Fig. 2c). In the historical simulation, inter-model differences in the mean ΔSSH are significantly related to the onshore (r_s_ = 0.84) and downwelling (r_s_ = −0.77) flows as expected (i.e. models with larger gradients tend to have stronger onshore flow and downwelling). The ΔSSH does not show a significant relationship with LC strength across the models, possibly since a similar amount of the onshore flow that the LC gains is downwelled into the LUC (Fig. 3). In contrast, there is a significant correlation with the LUC strength (Table 1), which may be because the LUC loses a large portion of transport via offshore transport (Fig. 3). We also find a negligible inter-model correlation between the ITF transport and ΔSSH (Table 1), which may indicate that inter-model differences in the ΔSSH are more strongly related to other local atmospheric forcing or processes occurring further south.

While there is a significant projected decrease of the zonal flows (Fig. 3), there is no accompanying significant projected decrease in the MMM ΔSSH (not shown). Despite this, much of the ΔSSH projected inter-model differences does explain inter-model differences in onshore (r_s_ = 0.81) and subsurface offshore flow (r_s_ = −0.59). A weaker onshore transport can drive either weaker downwelling or weaker LC transport. Given this and the strong historical correlation (r_s_ = −0.77), it is somewhat surprising that inter-model differences in the projected change of vertical transport are not significantly correlated with ΔSSH (Table 1). We do find models that project a smaller weakening of ΔSSH also tend to project a smaller LC weakening, although the relationship only becomes significant further south (Table 1). Contrary to expectation^7^, we find no inter-model relationship between the ITF and ΔSHH projected changes, despite the weak inter-annual relationship identified in two of the three sample models (Table 1).

### Western boundary transport

#### North East and South East Madagascar Currents (NEMC and SEMC)

As the broad westward flow of the South Equatorial Current approaches the east coast of Madagascar, it bifurcates into the northward flowing NEMC and the southward flowing SEMC^37^. The models simulate the two branches of flow, although there are considerable inter-model differences in the latitude of bifurcation as well as the depth and width of flow along the coast. As such, we only examine the NEMC and SEMC at 14°S and 24°S (the southern and northern latitude of Madagascar that is common to all models), respectively. Based on inspection of the annual mean velocity, we define the NEMC and SEMC as the net meridional transport in the upper 1500 m and, depending on the model, within 2° to 5° from the coast.

**Figure 4:**
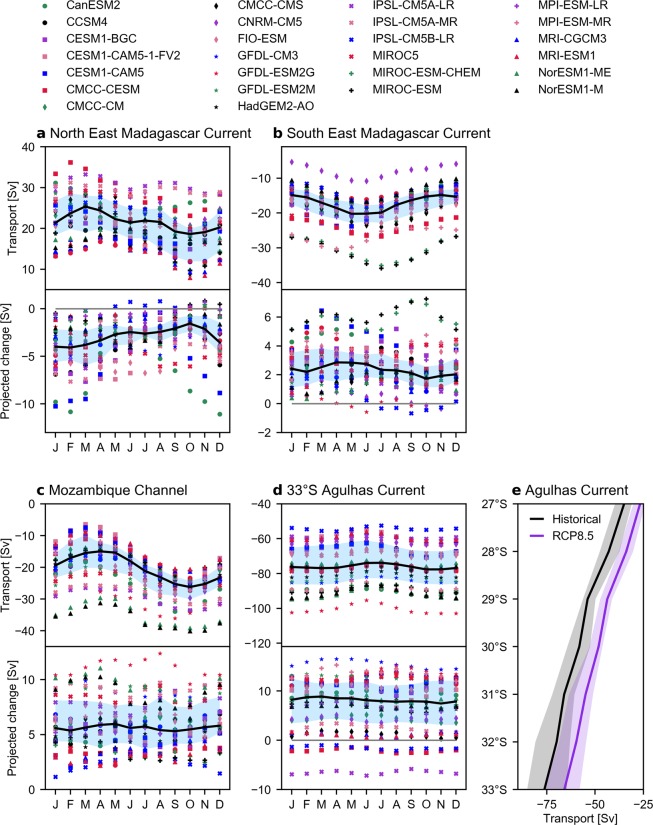
Western boundary historical transport and projected change. (**a**) North East Madagascar Current at 14°S monthly transport (Sv) in the historical scenario (top panel) and the projected change (bottom panel). (**b**) Same as (**a**), but for the South East Madagascar Current at 24°S. (**c**) Same as (**a**), but for the Mozambique Channel at 24°S. (**d**) Same as (**a**), but for the Agulhas Current at 33°S. (**e**) Agulhas Current annual multi-model median transport and associated interquartile range in the historical scenario (black) and RCP8.5 (purple). Shown in (**a–d**) are the multi-model median (black line), interquartile range (shaded blue) and individual models (markers; see legend) transport estimates. All projected changes are statistically significant at the 95% level.

In the historical scenario, the annual MMM transport of the NEMC is 21 Sv (18–24 Sv; Fig. 4a; top panel), lower than observational estimates which range from 27–48 Sv^38–40^ (Supplementary Table S2). The seasonal range is 6.7 Sv, where most models (≥19/28) simulate the strongest transport in February–April and the weakest transport in September–November (Fig. 4a; top panel). While there is limited observational data of the NEMC seasonal cycle for comparison, we find the simulated seasonal range is larger than the observational reports of 2 Sv^38^ or no discernible seasonality^39^. Further south, the poleward flowing SEMC transports −17 Sv (−15 to −20 Sv; Fig. 4b; top panel), which is a lower magnitude than observational estimates of −18 to −30 Sv^38,39,41–43^ (Supplementary Table S2). The SEMC transport seasonal timing is generally consistent across the models, with an annual range of 5.4 Sv (Fig. 4b; top panel).

All models project a weakening of the annual mean NEMC and SEMC transport, with MMM of −3.0 Sv (−2.1 to −4.0 Sv; 14% decrease) and 2.2 Sv (1.6 to 3.3 Sv; 13% decrease), respectively. There is a robust seasonality of the NEMC weakening across the models, where the greatest (least) weakening occurs around when the historical transport is strongest (weakest; Fig. 4a; bottom panel). Similarly, the MMM SEMC weakening is generally greatest when the historical transport is strongest, although the model agreement is weak (Fig. 4b; bottom panel). We also find models with stronger mean transports also project the greatest weakening for both the NEMC (r_s_ = −0.41, p = 0.03) and SEMC (r_s_ = −0.66, p < 0.001).

#### Mozambique Channel (MZC)

In the MZC, the CMIP5 models generally simulate continuous southward flow along the Mozambique coastline and, depending on the model, a number of alternating directions of flow across the channel. Based on the annual mean velocity, we define the transport through the MZC as the net meridional transport above 1500 m, between the coast of Mozambique and Madagascar at 24°S (although there is little change with latitudes).

The MMM transport through the channel is −20 Sv (−18 to −25 Sv; Fig. 4c; top panel), which is generally larger than observed transport ranging from −15 to −19 Sv^3,41,44,45^ (Supplementary Table S2). There is consistent model agreement on the transport seasonality, which is maximum in October (−26 Sv; 20/28 model agreement) and minimum around March–April (−15 Sv; 23/28 model agreement; Fig. 4c). This seasonality is similar to the observed and modelled transport maximum in September and minimum in March^44,46^, although there is a paucity of seasonal observations for comparison.

All models project the transport through the MZC will weaken, with MMM of 5.4 Sv (4.2–8.0 Sv), corresponding to a statistically significant decrease of 27%. We find there is no distinct seasonality of the projected change in the models (Fig. 4c; bottom panel). Similar to previous circulation features, models with larger mean historical transports tend to project stronger decreases (r_s_ = −0.74, p < 0.001).

#### Agulhas Current (AC)

We examine the strong western boundary AC from 27°S to 33°S where all models simulate southward flow adjacent to the African coast. We define the AC as the net meridional transport above 1500 m, although the simulated AC depth ranges from 1000 to 2000 m, and within 3° to 6° from the coast, depending on the model. The Agulhas Undercurrent that is apparent in observations^47,48^ is not evident in many of the models and so we did not examine it here.

The annual mean historical MMM transport of the AC increases as it flows southward along the coast, with MMM transport of −66 Sv (−61 to −72 Sv) at 31°S and −76 Sv (−65 to −85 Sv) at 33°S (Fig. 4e). The estimates are in good agreement with the observed transport of 70 Sv at 31°S^49^ and 77 Sv at 33°S^50^ (Supplementary Table S2). The MMM annual range of the AC ranges with latitude from 2.9 to 7.5 Sv and while there is no clear seasonal transport peak in the models, around 25 out of 28 models agree the transport is lowest in June–July (Fig. 4d). This seasonality is consistent with the observed March–April maxima and July–August minima^50,51^, with a similar seasonal range (3.8 Sv MMM at 33°S compared to ~5 Sv at 34°S^50^), suggested to be driven by local wind forcing and baroclinic processes^52^.

There is a projected slow down of the AC at all latitudes (Fig. 4e) and in all months (Fig. 4d; bottom panel). All models project a decrease of AC transport between 27–32°S, while only 25 of the 28 models project a weakening at 33°S. The largest magnitude of the projected change is at 31°S, with MMM of 10.6 Sv (5.9–11.8 Sv), corresponding to a 16% decrease. There is no clear seasonality in the projected change (Fig. 4d). As before, there is a significant inter-model relationship in which models that simulate larger AC transport in the historical scenario also project a larger weakening (e.g. r_s_ = −0.77 at 27°S and r_s_ = −0.47 at 33°S).

### Interior transport

#### Upper-ocean and Sverdrup transport

**Figure 5:**
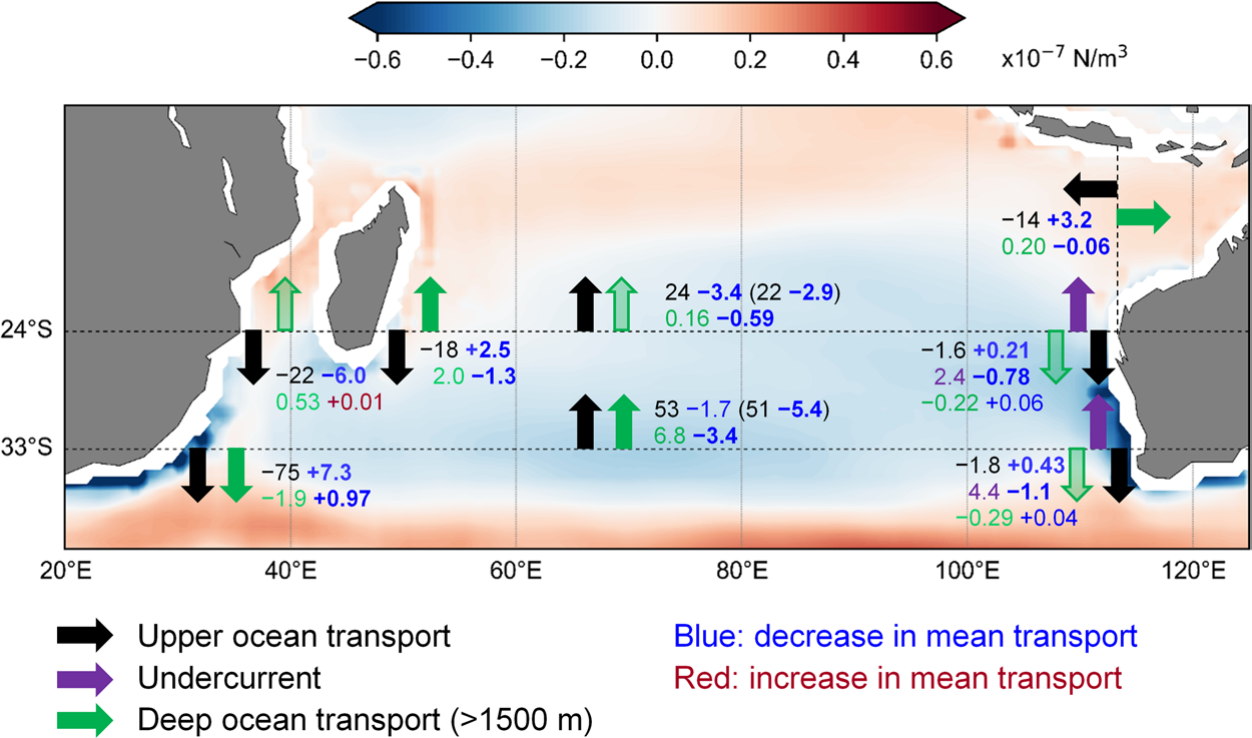
Mean and projected changes in transport. Multi-model mean transport (Sv) and projected changes at selected cross-sections. Black numbers and arrows represent upper-ocean transport shallower than 1500 m, excluding the Leeuwin Current (<200 m; black) and Leeuwin Undercurrent (200–1500 m; purple) off the west coast of Australia. Green numbers and arrows represent deep ocean transport (>1500 m; partially filled arrows represent poor inter-model agreement on direction). Northward and eastward historical transports are represented by positive values and arrows indicate the direction of historical transport, but not magnitude. Sverdrup transports (mean and projected change) are shown in brackets. Projected change values are coloured blue if weakening, red if strengthening and bold if significant at the 95% level. The multi-model mean wind stress curl projected change is shown in the background. To account for the different coastlines of each model, grid points where less than 50% of models simulate land are not shown. The multi-model mean wind stress curl in this figure does not include models FIO-ESM, MIROC-ESM-CHEM and MIROC-ESM due to errors in their archived wind stress curl.

The large poleward transport of water volume along the western boundary typically compensates for much of the equatorward upper-ocean interior basin transport. To help understand the drivers of the projected changes along the western boundary, we examine the upper-interior transport and wind stress curl, which can be related to the upper-interior transport via Sverdrup theory (see Methods). We examine the transport at two key locations: near the southern tip of Madagascar at 24°S and the southern entrance of the Indian Ocean at 33°S (Fig. 5). We define the upper-interior transport as the net meridional transport shallower than 1500 m between the offshore edges of the LC and the western boundary current (the SEMC at 24°S and the AC at 33°S). In most models, the flow between these latitudes is complicated by another jet of poleward flow further offshore of the AC.

In the historical scenario, the upper-interior transport decreases towards the north (Fig. 5; note that Fig. 5 shows MM mean transports, in order to provide a closed transport budget, while the transport values reported in the text below are MMM). Specifically, the MMM interior flow decreases from 54 Sv (47–60 Sv) at 33°S to 24 Sv (20–27 Sv) between Madagascar and Australia at 24°S. The MMM Sverdrup transport (inferred from the wind stress curl; see Methods) reproduced similar estimates of 50 Sv (45–55 Sv) at 33°S and 22 Sv (19–25 Sv) at 24°S.

The northward interior decrease is not related to the LC, as this flow has only a small change with latitude, nor the LUC as we found the northward LUC decrease is due to westward outflow into the interior South Indian Ocean (Fig. 3). Instead, the northward interior transport decrease is largely offset by the increase in the southward flowing AC (Fig. 4e). Inter-model differences in the upper-interior transport and inferred Sverdrup transports are significantly correlated (r_s_ = 0.81) at 24°S while further south at 33°S there is no statistically significant inter-model correlation, which indicates that factors unrelated to Sverdrup dynamics are needed to explain inter-model differences in the flow. For example, there are several large oceanic ridges (see Supplementary Figure S1 for the MM mean velocity profile) represented quite differently across the models that may alter the wind-driven flow near this region.

At 24°S, all models project a decrease in the upper-interior transport, with a MMM weakening of −3.8 Sv (−2.3 to −4.0 Sv), which is a statistically significant 16% decrease. At 33°S, the larger equatorward flow is not projected to weaken significantly, with MMM −2.0 Sv (+1.9 to −4.9 Sv). The zonally and vertically integrated upper-interior flow decreases at both latitudes. These changes, however, have a complicated zonal and vertical structure (Supplementary Fig. S1). For example, at 24°S the overall decrease includes a strengthening of the southward Ekman transport and at 33°S the decrease includes a strengthening of the zonally integrated subsurface northward flow (200 to 500 m; Supplementary Fig. S1). Despite the weak/inconsistent seasonality in the upper-interior historical transport, the projected weakening at 24°S has strong seasonality and is largest around June–July, while at 33°S the weakening is only significant from June to December (Supplementary Fig. S2).

Under RCP8.5, the mean wind stress curl and corresponding Sverdrup transport across both zonal cross-sections are projected to weaken (Fig. 5). All models project a decrease of Sverdrup transport at 24°S; the MMM decrease is 2.7 Sv, which is similar to, but larger than, the MMM 2.2 Sv decrease of the actual interior transport. A high inter-model correlation (r_s_ = 0.88, p < 0.001) between the Sverdrup and interior transport projected change at 24°S indicates that inter-model projected differences are strongly related to differences in projected wind changes. Similarly, at 33°S the Sverdrup transport is projected to decrease by all but one model, with MMM of 5.1 Sv (3.0–7.2 Sv; −10%). Interestingly, the projected change in wind forcing suggests a much greater weakening than the simulated interior change at 33°S and, as with the historical transport, there is no statistically significant inter-model correlation between the Sverdrup and interior transport projected changes.

#### Deep ocean transport

The deep ocean interior transport is defined as net meridional transport shallower than 1500 m and excluding the flow beneath the western and eastern boundary currents (see Fig. 5 for the MM mean transports of deep flow along the boundaries). The net direction of transport is not evident across the models (15/28 models simulate a net northward flow) because the meridional flow direction of deep waters at 24°S varies considerably across the basin. Consequently, the deep meridional MMM flow is weak with transport IQR of −0.92 to +1.1 Sv. At 33°S, the deep transport is consistently northward with a MMM of 5.6 Sv (5.0–7.3 Sv). In contrast to further north, the deep ocean transport at 33°S has a strong seasonality, with maximum in March (15 Sv) and minimum in August (−3.3 Sv; Supplementary Fig. S2).

The deep transport at 24°S is projected to change by a MMM of −0.56 Sv (+0.16 to −1.12 Sv). All but one of the 15 models that simulated northward flow project a reduction of deep transport and there are mixed projections in the remaining models. We found the MMM change is significant in November–April and September (Supplementary Fig. S2). The deep ocean transport flowing equatorward through 33°S is projected to decrease by MMM of −3.4 Sv (−2.3 to −4.5 Sv). This corresponds to a substantial 60% decrease, where 26 of 28 models agree on a weakening. The MMM weakening is greatest towards the start of the year (maximum in February; −4.5 Sv) and reaches a minimum in October (−2.3 Sv; Supplementary Fig. S2).

### Indian Ocean transport budget

**Figure 6:**
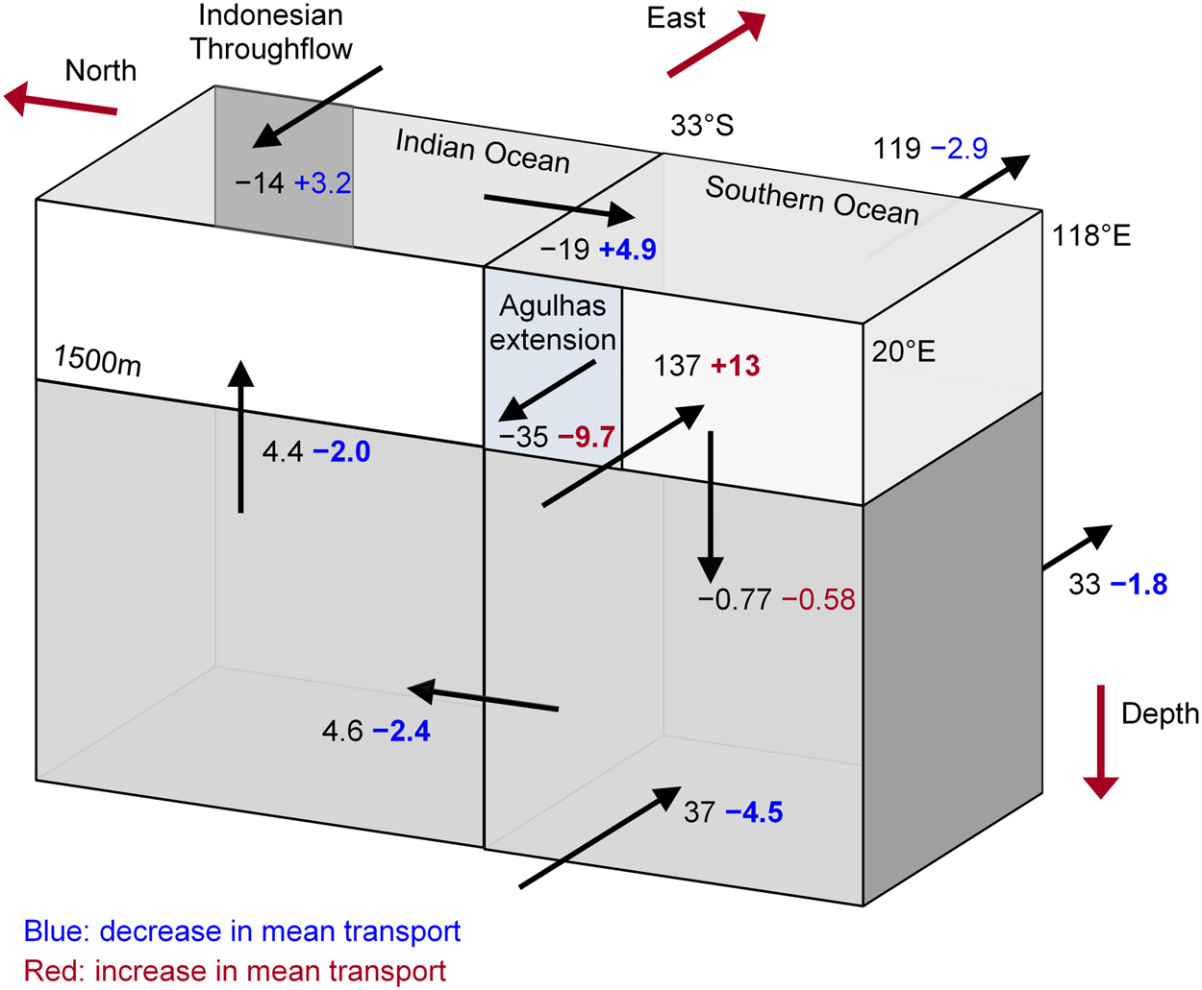
Mean and projected changes in transport in the Indian and Southern Oceans. Shown are multi-model mean transport (Sv) estimates in the historical scenario (black arrows and text) and the corresponding projected change (blue if weakening; red if strengthening; bold if significant at the 95% level). Northward, eastward and upward historical transports are represented by positive values and arrows indicate the direction of historical transport, but not magnitude.

To investigate overturning changes in the Indian Ocean, we create a transport budget using the net transport through the ITF and meridional transport across the 33°S southern Indian Ocean boundary and calculate vertical transport across 1500 m from continuity (Fig. 6; the negligible deep ocean transport through the ITF is not shown). There is a small MM mean error (−0.15 Sv) in the Indian Ocean transport budget, as the transports across the sections are not entirely consistent for a number of individual models. The error may be because we have not taken into account the net input of precipitation minus evaporation and, for some models, our ITF definition neglects some transport into the Indian Ocean. In the historical scenario, all models simulate net upward transport of waters across 1500 m for the entire Indian Ocean, with MM mean upwelling of 4.4 Sv (3.0 to 4.7 Sv; Fig. 6).

Under RCP8.5, all models project a decrease of upwelling, by MM mean of −2.0 Sv (−1.2 to −2.7 Sv), which corresponds to a substantial and statistically significant decrease of 47% (Fig. 6). There is an overall slow-down in the overturning circulation in the Indian Ocean (Fig. 6). That is, there is less deep water entering the Indian Ocean from the Southern Ocean and weaker upwelling which, in combination with less transport from the Pacific, reduces the basin-wide upper-ocean outflow (by a MM mean of 4.9 Sv, Fig. 6).

### Links to the Southern Ocean

We have identified considerable projected changes of South Indian Ocean circulation in the future and now examine the transport budget further south to help understand how the changes are related to the Southern Ocean. We integrate the net upper-ocean and deep zonal transport between Africa and Antarctica at 20°E and between Australia and Antarctica at 118°E (Fig. 6; MMM estimates are listed in Supplementary Table S3). Similar to the Indian Ocean transport budget, the Southern Ocean budget is not closed, with a MM error of 0.06 Sv.

In the transport budget, we distinguish the westward flow of the AC south of Africa at 20°E, defined as the AC extension, above 1500 m and north of 42°S. Most models (21/28) simulate mean transports that are less than 40 Sv while the remaining models (CCSM4, CESM1-BGC, CESM1-CAM5-1-FV2, CESM-CAM5, FIO-ESM, MPI-ESM-LR and MPI-ESM-MR) simulate transports that are greater than 60 Sv. Consequently, there is a skewed distribution of AC extension estimates and a large difference between the MMM of −28 Sv (IQR −16 to −46 Sv) and the MM mean of −35 Sv (Fig. 6). At 20°E, the Southern Ocean (minus the AC extension) transports a MM mean of 137 Sv (102–172 Sv) eastward in the upper 1500 m and 37 Sv (33–44 Sv) at lower depths (Fig. 6). South of Australia, the eastward flow of the Southern Ocean decreases to MM mean of 119 Sv (94–136 Sv) in the upper 1500 m and 33 Sv (30–38 Sv) at greater depths. Looking at the overall transport budget, the MM mean downwelling in the Southern Ocean is −0.77 Sv (+2.7 to −4.4 Sv; Fig. 6), although there is low model agreement, i.e., only 14/28 models simulate a net downward flux at 1500 m.

The AC extension is projected to increase in all but two models (CESM1-CAM5-1-FV2 and CESM1-CAM5) with MM mean strengthening of −9.7 Sv (−5.2 to −14 Sv), corresponding to a statistically significant 28% transport increase (Fig. 6). Despite a clear seasonality in the historical scenario, with an annual range of 5.2 Sv that peaks in February and troughs in June, there is no clear seasonality of the projected change. Nor is there a significant relationship between the strength of the AC extension in the historical and RCP8.5 scenario.

The upper-interior transport to the south of the AC extension is projected to increase in 26 out of 28 models, with a significant increase of 13 Sv (8–17 Sv; +9%; Fig. 6). In contrast, the deeper flow is projected to decrease significantly by a MM mean of −4.5 Sv (−1.7 to −7.4 Sv; −12%) and with 24/28 model agreement on the sign of the change (Fig. 6). South of Australia, the annual mean surface flow is not projected to change significantly, although the projected change is significant from February–April (Supplementary Fig. S4). The lack of a significant change masks the fact that overall, the northern part of the northern Antarctic Circumpolar Current is projected to weaken while the southern part of the Antarctic Circumpolar Current is projected to strengthen, indicative of a southward shift in the Antarctic Circumpolar Current (Supplementary Fig. S3). The deeper flow is projected to weaken significantly, but by a smaller MM mean of −1.8 Sv (+0.7 to −3.8 Sv; −6%) and relatively low model agreement (18/28) than the deeper flow south of Africa (Fig. 6). Unlike the weakening of deep flow at 20°E that is significant throughout the year, the weakening at 118°E is significant from August to April (Supplementary Fig. S4). The mean downwelling in the Southern Ocean is not projected to change significantly (Fig. 6), indicating that projected changes in the surface and deep Southern Ocean transport are not related through changes in vertical transport.

## Summary

In this paper, we used an ensemble of 28 CMIP5 models to examine South Indian Ocean circulation in the 20^th^ century and the projected change for the second half of the 21^st^ century due to ‘business as usual’ greenhouse gas emission increases. We examined the ensemble median (or mean) and model spread of long-term mean seasonal and annual volume transports of large-scale circulation features and compared them to observations. Additionally, we determined the statistical significance of projected changes and explored mechanisms driving variability and projected changes.

The input of Pacific waters into the Indian Ocean via the Indonesian Throughflow (ITF), is projected by the models to decrease by about one quarter, in agreement with previous studies^7,53,54^. Despite the strong mean ITF seasonality, we found the projected ITF weakening has no clear seasonality (Fig. 1a), although a previous study reported some seasonality in the ITF projected change^7^. The projected ITF reduction is linked to the slow down of Pacific deep upwelling and weakening of Pacific overturning circulation^53,54^.

While there are large inter-model differences, most models agree that the Leeuwin Current (LC) will weaken (2% to 11%), suggesting our results provide a meaningful indication of the sign of the future change (Fig. 2a). While the CMIP5 models are unable to capture mesoscale dynamical processes, the projected LC decrease is consistent with the projected weakening found in an eddy-resolving ocean model forced by bias-corrected atmospheric output from an atmosphere-ocean coupled model subject to the Special Report on Emissions Scenarios A1B scenario^7^. We found that the projected LC weakening has a distinct latitude-dependent seasonality (Fig. 2b,c). The seasonality of the change towards the south agrees with the eddy-permitting model^7^ and further north the projected change was not previously examined. The models consistently project a significant weakening of the subsurface Leeuwin Undercurrent (LUC) of up to 17% (Fig. 2d), with no clear seasonality of the projected changes (Fig. 2e,f).

We show that the LC system transport changes between 26°S and 32°S are related to a 41% reduction of the onshore transport into the LC and a 24% reduction of LC downwelling (Fig. 3). The small projected strengthening of the southerly winds, which is thought to influence the LC transport on seasonal timescales, is consistent with the LC slow down. However, differences in the wind projections across the models could not explain inter-model differences in upwelling or LC strength on these multi-decadal timescales (Table 1). The annual mean meridional difference in sea surface height (ΔSSH) was not projected to change significantly, although there is a small significant weakening in August and September. While the overall reduction in the LC system cannot be linked to the ΔSSH, the model differences in the projected ΔSSH change can explain a large part of the inter-model differences in the zonal and LC transports near 32°S (Table 1). Despite finding an inter-annual relationship between ITF transport and the ΔSSH, on longer timescales, we found that inter-model differences in the projected ITF strength could not explain the differences in the ΔSSH change (Table 1). As such, other mechanisms are likely to be driving the ΔSSH change and the subsequent LC system change. For example, there may be a change in heat content further south off the Western Australia coast.

In the south-western Indian Ocean, the models project a consistent weakening of transport via the North East Madagascar Current (NEMC), South East Madagascar Current (SEMC) and Mozambique Channel (MZC) by 14%, 13% and 27%, respectively. These weaker transports are partly offset by the 16% reduction of northward upper-ocean interior transport at 24°S (Fig. 5). We find that the projected decrease of wind stress curl plays a primary role in explaining inter-model differences in the upper-interior transport between Madagascar and Australia. Further analysis using the Island Rule^55^ could illuminate the projected wind-driven response of flow through the MZC. Additionally, the NEMC, SEMC and MZC transport weakening is potentially related to changes of the westward South Equatorial Current and its bifurcation latitude^56,57^.

Further south, the models project a substantial decrease of Agulhas Current (AC) transport by 11–23% (Fig. 4e). The AC was also projected to decrease in a previous idealised study^14^ using an eddy-permitting ocean model forced by an intensification of southern hemisphere westerlies and southward shift of the wind stress curl zero line. In that case, the AC weakening was associated with a 10% reduction in wind stress curl^14^. Despite finding a similar projected multi-model median wind stress curl weakening, we did not find a corresponding significant upper-ocean decrease at 33°S (Fig. 5) or inter-model relationship between changes in transport and wind stress curl.

The projected decrease of deep Southern Ocean transport south of Africa is associated with weaker deep, northward flow into the Indian Ocean (Fig. 6). The combined reduction of overturning circulation in the Indian Ocean and transport through the ITF (Fig. 6) is consistent with the slow down of western boundary circulation.

There are several key uncertainties to take into consideration when interpreting our projections. Firstly, the wind locations in the CMIP5 models are systematically biased, with the westerlies over the Southern Ocean located too far equatorward^58–60^. If the projected changes are state-dependent, the projected circulation changes are likely to be systematically influenced. A second important limitation is that mesoscale processes that are parameterised in CMIP5 models could be critical to realistically simulating future oceanic changes, especially for regions of energetic circulation of the Indian Ocean including the Agulhas Current system^61^. Despite this, our results reveal qualitatively similar changes to those from previous studies using individual high-resolution models (e.g. the ITF^54^, LC^7^ and AC^14^). It is also clear that the paucity of *in situ* and other measurements hamper our ability to validate aspects of the models’ circulation and the dynamical drivers. Comprehensive and sustained observational data is needed to properly understand the response of South Indian Ocean circulation to a changing climate.

Overall, the CMIP5 models are able to simulate many aspects of the complex South Indian Ocean circulation, although there are considerable quantitative inter-model differences. Under RCP8.5 forcing, the models project a number of robust future changes, including a slow down of almost all South Indian Ocean circulation, which may have important consequences for regional weather and marine resources.

## Supplementary information

Supplementary Information

## Data Availability

All CMIP5 model output is available at https://esgf-node.llnl.gov/search/cmip5/. Data processing and analysis used Climate Data Operators, Python and the SciPy library. All scripts, processed transport files for the CMIP5 models and integration boundary definitions are available from the corresponding author on request.
