# Supplementary material for: Projected slow down of South Indian Ocean circulation
Source: Sci Rep. 2019 Nov 27;9:17705. doi: 10.1038/s41598-019-54092-3 (PMC6881323; doi:10.1038/s41598-019-54092-3)
Supplement: Supplementary file 1 — Supplementary Information [file 41598_2019_54092_MOESM1_ESM.pdf]

Scientific Reports

Supplementary Information for

Projected slow down of South Indian Ocean circulation

Annette Stellema[1,*], Alex Sen Gupta[1] and Andréa S. Taschetto[1]

[1]Climate Change Research Centre and Australian Research Council Centre of Excellence for Climate Extremes, University of New South Wales, Sydney, Australia

*Correspondence: a.stellema@unsw.edu.au

Contents of this file

Table S1 2
Table S2 3
Table S3 5
Figure S1 8
Figure S2 9
Figure S3 10
Figure S4 11
References 12

Table S1. CMIP5 models used in this study. Shown are the model names, institute and country of origin, the horizontal resolution for the ocean and atmosphere components, the number of ocean vertical levels and the reference.

Model	Institute	Resolution (°lat × °lon)		Ocean vertical levels	Reference
		Atmosphere	Ocean		
ACCESS1.0	CSIRO-BOM, Australia	1.9 × 1.2	1.0 × 1.0	50	[1]
ACCESS1.3	CSIRO-BOM, Australia	1.9 × 1.2	1.0 × 1.0	50	[1]
CanESM2	CCCMA, Canada	2.8 × 2.8	1.4 × 0.9	40	[2]
CCSM4	NCAR, USA	1.2 × 0.9	1.1 × 0.6	60	[3]
CESM1-BGC	NSF-DOE-NCAR, USA	1.2 × 0.9	1.1 × 0.6	60	[4]
CESM1-CAM5	NSF-DOE-NCAR, USA	1.2 × 0.9	1.1 × 0.6	60	[5]
CESM1-CAM5.1.FV2	NSF-DOE-NCAR, USA	2.0 × 1.9	1.1 × 0.6	60	[5]
CMCC-CESM	CMCC, Italy	3.7 × 3.7	2.0 × 1.9	31	[6]
CMCC-CM	CMCC, Italy	0.7 × 0.7	2.0 × 1.9	31	[7]
CMCC-CM5	CMCC, Italy	1.9 × 1.9	2.0 × 1.9	31	[6]
CNRM-CM5	CNRM-CERFACS, France	1.4 × 1.4	1.0 × 0.8	42	[8]
FIO-ESM	FIO, SOA, China	2.8 × 2.8	1.1 × 0.6	40	[9]
GFDL-CM3	NOAA, GFDL, USA	2.5 × 2.0	1.0 × 1.0	50	[10]
GFDL-ESM2G	NOAA, GFDL, USA	2.5 × 2.0	1.0 × 1.0	50	[10]
GFDL-ESM2M	NOAA, GFDL, USA	2.5 × 2.0	1.0 × 1.0	50	[10]
HadGEM2-AO	NIMR-KMA, Korea	1.9 × 1.2	1.0 × 1.0	40	[11]
IPSL-CM5A-LR	IPSL, France	3.7 × 1.9	2.0 × 1.9	31	[12]
IPSL-CM5A-MR	IPSL, France	3.7 × 1.9	2.0 × 1.9	31	[12]
IPSL-CM5B-LR	IPSL, France	2.5 × 1.3	1.6 × 1.4	31	[12]
MIROC5	JAMSTEC, Japan	1.4 × 1.4	1.6 × 1.4	50	[13]
MIROC-ESM	JAMSTEC, Japan	2.8 × 2.8	1.4 × 0.9	44	[14]
MIROC-ESM-CHEM	JAMSTEC, Japan	2.8 × 2.8	1.4 × 0.9	44	[14]
MPI-ESM-LR	MPI-M, Germany	1.9 × 1.9	1.5 × 1.5	40	[15]
MPI-ESM-MR	MPI-M, Germany	1.9 × 1.9	0.4 × 0.4	40	[15]
MRI-CGCM3	MRI, Japan	1.1 × 1.1	1.0 × 0.5	51	[16]
MRI-ESM1	MRI, Japan	1.1 × 1.1	1.0 × 0.5	51	[16]
NorESM1-ME	NCC, Norway	2.5 × 1.9	1.1 × 0.6	70	[17]
NorESM1-M	NCC, Norway	2.5 × 1.9	1.1 × 0.6	70	[17]

Table S2. Indicative volume transport estimates from observational data. Including estimated transport of the Indonesian Throughflow, Leeuwin Current, Leeuwin Undercurrent, North East Madagascar Current, South East Madagascar Current, Mozambique Channel and Agulhas Current. If available, the observation location, mean transport (1 Sv ≡ 10^6 m^3 s^{-1}), seasonal transport maximum and minimum (and time of year), reference depth and data source is given. Positive values indicate northward or eastward transport. Note that this list is generally limited to observational estimates with similar measurement locations and reference depths as used in this study and long-term measurements (if available).

Location	Mean Transport (Sv)	Transport maximum (Sv)	Transport minimum (Sv)	Reference depth	Source
Indonesian Throughflow					
—	−10	—	—	—	Derived from wind observations[18]
—	−13.1 to −13.5	—	—	—	Derived from wind observations[19]
—	−16 ± 5	—	—	< 27.72 kg m^{-3}	Hydrographic data[20]
—	−15.0 (−10.1 to −18.7)	−17.9 (July)	(Feb)	Full depth	Current meter moorings[21,22]
Leeuwin Current					
22	−4[a]	—	—	> 150 m	Hydrographic data[23]
22°S	−0.3	—	—	200 m	Hydrographic data[24]
25°S	—	−4.2 (Sep & Mar)[a]	−1.8 (Jan–Feb)[a]	300 m	Current meter moorings[25]
29.5°S	—	−6.8 (Jun)[a]	−1.4 (Jan–Feb)[a]	300 m	Current meter moorings[25]
32°S	−3.4[a]	−5 (June–July)[a]	−2 to −3 (Jan–Feb)[a]	300 m	Derived from temperature data[26]
34°S	—	−5.1 (Jun) [a]	−1.3 (Jan–Feb)[a]	300 m	Current meter moorings[25]
34°S	−1.5	—	—	200 m	Hydrographic data[24]
22–34°S	—	1.6 (May)	0.6 (Oct)	200 m	Hydrographic data[24]
25–30°S	—	4.5 E (Sep)[a]	2.2 E (Jan–Feb & Aug)[a]	300 m	Current meter moorings[25]
Leeuwin Undercurrent					
22.5	5[a]	—	—	> 150 m	Hydrographic data[23]
22°S	1.7	—	—	200–800 m	Hydrographic data[24]
28°S	3.4	—	—	200–800 m	Hydrographic data[24]
22–34°S	—	2.2 (Oct)	1.7 (May)	200–800 m	Hydrographic data[24]

Table S2 (continued)

North East Madagascar Current					
12°S	29.6[a]	—	—	1100 dbar	Ship drifts and near surface hydrography[27]
12°S	26.9 ± 9.4[a]	Negligible	Negligible	1100 m	Current meter moorings[28]
12°S	48 ± 4[a]	—	—	1100 m	Hydrographic data[29]
South East Madagascar Current					
23°S	−20.6	—	—	1170 dbar	Ship drifts and near surface hydrography[27]
23°S	−20.3 ± 6.6[a]	Negligible	Negligible	1170 dbar	Current meter moorings[28]
23°S	−17.8 ± 6.8	—	—	1500 m	Current meter moorings and satellite altimetry[30]
25°S	−20[a]	—	—	> 2000 m	Hydrographic and float data[31]
25°S	−30[a]	—	—	1100 m	Hydrographic and satellite altimetry data[32]
Mozambique Channel					
17°S	−15[a]	—	—	1500 m	Hydrographic data[33]
17°S	−16.7 ± 3.1	(Sep)	(Mar)	1500 m	Current meter moorings[34]
24°S	−19[a]	—	—	2000 m	Hydrographic and float data[35]
25°S	−17.7[a]	—	—	> 2000 m	Hydrographic and float data[31]
Agulhas Current					
31°S	−69.7 ± 4.3	—	—	2400 m	Current meter moorings[36]
32°S	−48 to −93[a]	—	—	Full depth	Hydrographic data[37–41]
~32°S	−76 ± 2[a]	—	—	> 2000 m	Hydrographic data[31]
34°S	−77 ± 4	(Mar−Apr)	(Aug)	3100 m	Current meter moorings and satellite altimeter[42]
—	—	(Dec–Feb)	(Jun–Aug)	—	Satellite altimetry[43]
[a]Based on less than one year of data.					

Table S3. Transport values for the 20th century mean (20C), absolute change (Δ) and percent change (%Δ). Annual mean transports (Sv) for individual models, multi-model (MM) mean and median. Negative values in 20C indicate southward or westward transport, where a corresponding positive Δ indicates a transport decrease.

	Indonesian Throughflow			Leeuwin Current at 32°S			Leeuwin Undercurrent at 32°S			North East Madagascar Current at 14°S			South East Madagascar Current at 24°S			Mozambique Channel at 24°S		
	**20C**	**Δ**	**%Δ**	**20C**	**Δ**	**%Δ**	**20C**	**Δ**	**%Δ**	**20C**	**Δ**	**%Δ**	**20C**	**Δ**	**%Δ**	**20C**	**Δ**	**%Δ**
ACCESS1-0	−13	3.8	−29	−2.5	0.5	−20	3.9	−0.7	−17	14	−3.3	−22	−15	1.6	−11	−21	6.1	−29
ACCESS1-3	−15	3.5	−23	−2.7	1.1	−40	3.9	−1.0	−25	14	−3.6	−26	−15	3.1	−21	−20	3.7	−18
CanESM2	−18	3.5	−19	−1.6	0.1	−3	4.6	−0.3	−8	26	−6.7	−26	−18	3.3	−18	−26	5.3	−21
CCSM4	−12	2.6	−21	−2.1	−0.0	2	3.7	−0.3	−8	19	−4.0	−21	−16	2.9	−18	−17	5.3	−32
CESM1-BGC	−12	2.6	−21	−2.1	−0.0	0	3.6	−0.2	−7	19	−4.0	−21	−17	3.4	−20	−16	5.4	−33
CESM1-CAM5-1-FV2	−11	2.6	−23	−2.1	−0.2	12	2.9	−0.2	−7	22	−3.9	−18	−19	2.8	−14	−15	5.1	−33
CESM1-CAM5	−12	3.2	−27	−2.3	0.1	−3	4.0	−0.4	−11	21	−5.6	−27	−19	4.3	−23	−17	4.4	−25
CMCC-CESM	−11	1.1	−10	−2.5	0.5	−19	7.3	−1.0	−14	29	−3.2	−11	−24	2.9	−12	−17	3.4	−20
CMCC-CM	−11	1.4	−13	−2.0	0.1	−5	6.0	−0.4	−7	23	−3.0	−13	−20	2.1	−10	−19	6.1	−33
CMCC-CMS	−12	1.4	−12	−2.3	0.3	−12	6.8	−0.6	−9	24	−2.2	−9	−20	1.6	−8	−21	6.0	−28
CNRM-CM5	−11	2.0	−18	−2.3	0.3	−15	3.2	−0.4	−14	26	−2.6	−10	−8	0.5	−6	−22	4.7	−22
FIO-ESM	−16	4.0	−26	−0.9	−0.1	14	3.7	−0.4	−11	22	−5.3	−24	−19	3.7	−19	−24	6.8	−28
GFDL-CM3	−14	4.5	−32	−1.9	0.3	−15	4.2	−0.5	−12	23	−4.6	−20	−17	1.7	−10	−20	8.9	−44
GFDL-ESM2G	−22	5.8	−27	−1.8	−0.0	3	2.9	0.1	5	24	−2.2	−9	−17	0.2	−1	−31	11.1	−36
GFDL-ESM2M	−18	4.7	−26	−1.9	0.1	−3	3.7	−0.2	−7	25	−2.9	−11	−16	1.3	−8	−26	9.1	−35
HadGEM2-AO	−13	3.5	−28	−1.5	0.3	−24	6.2	−0.5	−8	16	−2.5	−15	−17	2.2	−13	−19	4.5	−24
IPSL-CM5A-LR	−13	1.1	−9	−2.4	0.0	−0	7.6	−0.9	−12	31	−3.5	−11	−18	1.3	−8	−30	7.8	−26
IPSL-CM5A-MR	−13	1.9	−14	−2.6	0.1	−2	7.3	−1.3	−17	30	−5.2	−18	−18	2.1	−12	−28	9.2	−33
IPSL-CM5B-LR	−10	1.2	−12	−2.5	0.3	−14	6.6	−0.2	−3	23	−0.3	−1	−14	0.4	−3	−21	2.4	−11
MIROC5	−14	4.1	−30	−1.2	0.1	−8	4.5	−0.9	−20	24	−5.0	−21	−23	3.7	−16	−24	8.5	−35
MIROC-ESM-CHEM	−18	4.1	−22	−2.5	1.1	−43	5.3	−1.1	−20	18	−1.1	−6	−30	5.9	−19	−18	3.5	−20
MIROC-ESM	−19	3.7	−20	−2.6	1.0	−38	5.3	−0.9	−18	18	−0.9	−5	−31	6.1	−20	−18	3.5	−20
MPI-ESM-LR	−14	4.5	−31	−4.6	0.2	−4	9.8	−0.1	−1	2	−0.4	−24	−18	3.1	−17	−18	5.4	−30
MPI-ESM-MR	−14	4.3	−30	−2.8	0.2	−6	5.7	−0.5	−9	21	−3.0	−14	−27	4.0	−15	−19	6.6	−34
MRI-CGCM3	−12	3.0	−24	−2.1	0.3	−14	4.0	−0.5	−12	14	−2.0	−14	−16	2.1	−13	−15	3.5	−23
MRI-ESM1	−12	2.6	−22	−2.0	0.2	−11	3.9	−0.3	−9	14	−1.4	−11	−15	1.7	−12	−15	2.9	−20
NorESM1-ME	−20	4.5	−22	−2.3	−0.1	3	3.1	−0.5	−16	19	−1.7	−9	−14	1.3	−9	−35	9.6	−27
NorESM1-M	−21	4.1	−20	−2.3	−0.1	4	3.1	−0.4	−14	18	−2.4	−13	−13	1.7	−13	−36	8.9	−25
**MM Mean**	**−14**	**3.2**	**−22**	**−2.2**	**0.2**	**−10**	**4.9**	**−0.5**	**−11**	**21**	**−3.1**	**−15**	**−18**	**2.5**	**−14**	**−22**	**6.0**	**−28**
**MM Median**	**−13**	**3.5**	**−26**	**−2.3**	**0.1**	**−6**	**4.1**	**−0.5**	**−11**	**21**	**−3.0**	**−14**	**−17**	**2.2**	**−13**	**−20**	**5.4**	**−27**

Table S3 (continued)

	Agulhas Current at 33°S			Agulhas Extension at 20°E			Interior transport at 24°S (0-1500m)			Sverdrup transport at 24°S			Interior transport at 33°S (0-1500m)			Sverdrup transport at 33°S		
	**20C**	**Δ**	**%Δ**	**20C**	**Δ**	**%Δ**	**20C**	**Δ**	**%Δ**	**20C**	**Δ**	**%Δ**	**20C**	**Δ**	**%Δ**	**20C**	**Δ**	**%Δ**
ACCESS1-0	−76	12.4	−16.3	−37	−12.4	34	23	−1.9	−9	25	−1.5	−6	60	−6.3	−10.5	55	−7.3	−13
ACCESS1-3	−78	12.4	−15.9	−38	−8.3	22	20	−1.5	−8	22	−1.2	−5	59	−6.7	−11.2	51	−5.2	−10
CanESM2	−90	9.6	−10.7	−17	−15.1	91	23	−3.9	−17	20	−3.3	−16	65	−4.1	−6.3	64	−8.8	−14
CCSM4	−66	11.2	−17.1	−72	−2.9	4	20	−3.8	−19	16	−1.7	−11	48	−5.1	−10.7	42	−5.0	−12
CESM1-BGC	−66	11.2	−16.8	−71	−3.3	5	20	−4.2	−20	16	−2.4	−15	48	−4.8	−9.9	43	−5.5	−13
CESM1-CAM5-1-FV2	−59	8.7	−14.7	−76	1.1	−1	25	−3.7	−15	19	−2.3	−12	44	−2.7	−6.2	36	−2.3	−6
CESM1-CAM5	−66	11.6	−17.6	−75	1.0	−1	24	−3.9	−16	19	−2.5	−13	49	−4.7	−9.7	43	−5.0	−12
CMCC-CESM	−76	−2.1	2.7	−16	−7.1	45	28	−2.8	−10	25	−2.9	−12	54	5.3	9.9	68	−5.7	−8
CMCC-CM	−75	4.4	−5.8	−16	−9.9	61	24	−4.0	−17	25	−3.8	−16	54	−0.6	−1.1	55	−6.4	−12
CMCC-CMS	−77	1.2	−1.6	−15	−10.2	66	26	−3.3	−13	28	−3.4	−12	54	2.7	5.0	59	−6.2	−10
CNRM-CM5	−62	4.3	−7.0	−30	−4.9	17	20	−2.4	−12	18	−2.2	−12	48	−0.9	−2.0	46	−1.5	−3
FIO-ESM	−69	10.7	−15.5	−62	−8.2	13	27	−4.8	−18	24	−4.5	−18	45	−3.0	−6.6	54	−4.9	−9
GFDL-CM3	−84	15.5	−18.4	−31	−14.2	46	22	−3.4	−15	22	−2.3	−11	64	−7.5	−11.8	57	−8.2	−14
GFDL-ESM2G	−100	13.2	−13.1	−27	−2.6	10	24	−2.3	−10	23	−2.0	−9	69	−5.8	−8.4	46	−2.7	−6
GFDL-ESM2M	−82	12.8	−15.5	−15	−18.0	118	21	−2.3	−11	21	−2.5	−12	59	−4.5	−7.7	48	−4.3	−9
HadGEM2-AO	−81	7.7	−9.5	−40	−13.1	32	20	−1.6	−8	17	−1.8	−11	59	−1.0	−1.7	55	−7.2	−13
IPSL-CM5A-LR	−60	−6.5	11.0	−9	−10.0	114	32	−5.0	−16	27	−5.4	−20	36	10.2	28.3	68	−9.4	−14
IPSL-CM5A-MR	−68	2.5	−3.7	−14	−13.5	94	30	−6.1	−21	25	−6.1	−24	44	3.5	7.9	66	−13.9	−21
IPSL-CM5B-LR	−54	−1.5	2.8	−9	−1.7	19	25	−0.9	−4	22	−1.3	−6	39	2.9	7.5	51	0.2	0
MIROC5	−77	12.5	−16.2	−24	−16.7	70	20	−3.8	−19	18	−3.2	−17	48	−11.9	−24.6	48	−7.5	−16
MIROC-ESM-CHEM	−88	7.7	−8.7	−24	−14.5	60	28	−4.0	−14	35	−3.6	−10	65	−2.3	−3.5	44	−3.0	−7
MIROC-ESM	−89	6.8	−7.6	−23	−16.2	70	28	−4.5	−16	36	−4.5	−13	66	−1.8	−2.7	45	−2.8	−6
MPI-ESM-LR	−60	6.7	−11.3	−82	−14.0	17	42	−5.0	−12	27	−3.3	−12	52	−0.5	−1.0	54	−2.8	−5
MPI-ESM-MR	−88	13.6	−15.5	−71	−26.1	37	33	−4.7	−14	27	−4.4	−16	70	−7.0	−10.0	64	−9.4	−15
MRI-CGCM3	−59	1.2	−2.1	−18	−8.9	49	17	−1.7	−10	12	−1.3	−11	41	3.0	7.2	48	−5.1	−11
MRI-ESM1	−59	1.1	−1.8	−18	−9.2	50	16	−1.1	−6	11	−0.9	−8	41	2.9	6.9	48	−4.5	−10
NorESM1-ME	−92	8.7	−9.5	−32	−5.2	17	24	−3.8	−16	19	−3.1	−16	59	0.3	0.6	39	−3.0	−8
NorESM1-M	−91	6.8	−7.5	−28	−8.0	29	24	−4.0	−17	20	−3.4	−18	57	1.6	2.8	40	−3.5	−9
**MM Mean**	**−75**	**7.3**	**−9.8**	**−35**	**−9.7**	**28**	**24**	**−3.4**	**−14**	**22**	**−2.9**	**−13**	**53**	**−1.7**	**−3.3**	**51**	**−5.4**	**−10**
**MM Median**	**−76**	**8.2**	**−10.8**	**−28**	**−9.6**	**35**	**24**	**−3.8**	**−16**	**22**	**−2.7**	**−12**	**54**	**−2.0**	**−3.7**	**50**	**−5.1**	**−10**

Table S3 (continued)

	Interior transport at 24°S (1500m+)			Interior transport at 33°S (1500m+)			Southern Ocean at 20°E (0-1500m)			Southern Ocean at 20°E (1500m+)			Southern Ocean at 118°E (0-1500m)			Southern Ocean at 118°E (1500m+)		
	**20C**	**Δ**	**%Δ**	**20C**	**Δ**	**%Δ**	**20C**	**Δ**	**%Δ**	**20C**	**Δ**	**%Δ**	**20C**	**Δ**	**%Δ**	**20C**	**Δ**	**%Δ**
ACCESS1-0	−0.4	0.2	−59	4.7	−3.0	−63	118	4.8	4.1	34	−6.2	−18	135	−2.0	−1.5	30	−3.6	−11.8
ACCESS1-3	0.3	0.7	240	5.1	−3.0	−59	137	−5.2	−3.8	49	−15.2	−31	156	−12.4	−7.9	44	−12.2	−27.4
CanESM2	0.2	−0.8	−444	7.3	−3.6	−49	127	6.1	4.8	29	−2.3	−8	142	2.0	1.4	31	−1.5	−4.9
CCSM4	−1.2	−0.2	19	4.6	−6.0	−132	126	7.2	5.7	48	−6.2	−13	146	−1.5	−1.0	40	0.0	0.0
CESM1-BGC	−0.8	−0.3	38	5.3	−6.2	−118	120	6.9	5.7	49	−7.8	−16	141	−2.2	−1.5	41	−1.3	−3.2
CESM1-CAM5-1-FV2	−1.7	0.3	−18	1.9	−4.3	−222	123	9.6	7.8	38	−2.7	−7	138	3.3	2.4	34	1.1	3.1
CESM1-CAM5	−2.2	0.5	−21	4.2	−5.3	−127	118	10.7	9.1	36	−2.9	−8	135	3.3	2.4	31	1.3	4.2
CMCC-CESM	1.6	−0.3	−18	6.8	−1.5	−22	75	1.3	1.7	33	−4.4	−13	94	−0.6	−0.6	26	−3.8	−14.6
CMCC-CM	−0.8	0.4	−52	5.0	−2.6	−51	77	3.8	4.9	27	−3.1	−12	88	1.0	1.1	26	−1.9	−7.2
CMCC-CMS	0.7	−0.4	−52	6.0	−2.4	−41	81	3.6	4.5	30	−2.5	−8	100	−1.3	−1.3	22	1.0	4.4
CNRM-CM5	−3.3	0.6	−17	1.4	−1.2	−88	72	6.4	8.8	16	−2.1	−14	81	3.6	4.5	19	−1.4	−7.5
FIO-ESM	1.0	−0.9	−85	10.2	−6.6	−65	109	−6.3	−5.7	49	−15.1	−31	133	−17.2	−13.0	42	−7.8	−18.6
GFDL-CM3	−2.8	0.4	−14	5.6	−4.2	−75	115	1.1	1.0	41	−9.2	−23	132	−6.9	−5.2	40	−6.1	−15.4
GFDL-ESM2G	4.8	−1.9	−39	8.7	−4.3	−50	69	8.3	12.0	39	−7.4	−19	94	−2.1	−2.3	35	−1.6	−4.6
GFDL-ESM2M	5.9	−1.5	−25	7.4	−4.4	−59	93	7.5	8.1	36	−8.6	−24	111	−1.0	−0.9	34	−4.7	−13.5
HadGEM2-AO	2.6	−1.8	−68	8.1	−2.4	−30	118	−6.3	−5.3	46	−7.6	−17	132	−13.6	−10.4	45	−3.8	−8.4
IPSL-CM5A-LR	0.7	−1.8	−278	5.3	0.6	10	62	10.5	16.8	35	2.4	7	85	5.6	6.6	23	5.7	24.9
IPSL-CM5A-MR	0.6	−2.0	−328	6.7	−2.1	−30	70	11.0	15.7	42	0.0	0	94	5.6	6.0	29	3.2	11.1
IPSL-CM5B-LR	−2.6	0.1	−5	4.7	0.2	4	73	1.8	2.5	17	−0.2	−1	83	0.2	0.2	15	0.2	1.1
MIROC5	1.3	−1.9	−149	9.3	−5.9	−64	101	−0.9	−0.9	51	−11.2	−22	127	−14.3	−11.3	41	−1.7	−4.2
MIROC-ESM-CHEM	−0.3	−0.8	250	5.5	−3.3	−59	136	0.8	0.6	40	−4.5	−11	156	−5.8	−3.7	37	−1.4	−3.8
MIROC-ESM	−0.3	−0.8	271	5.6	−3.2	−57	134	0.1	0.0	40	−4.0	−10	155	−6.4	−4.1	36	−0.6	−1.7
MPI-ESM-LR	−2.4	−0.3	12	4.9	−2.7	−54	114	5.8	5.1	42	−2.7	−6	134	−1.9	−1.4	35	0.6	1.7
MPI-ESM-MR	0.8	−0.8	−101	6.1	−4.1	−67	115	3.3	2.9	36	−4.2	−11	130	−2.7	−2.1	36	−2.8	−7.7
MRI-CGCM3	1.8	−1.1	−60	5.6	−1.9	−34	88	−13.4	−15.2	30	−0.0	−0	95	−10.8	−11.3	36	−6.2	−17.2
MRI-ESM1	1.5	−1.0	−67	5.5	−1.9	−35	85	−12.9	−15.1	29	0.3	1	92	−10.2	−11.0	35	−5.7	−16.3
NorESM1-ME	0.3	−1.3	−395	19.9	−6.3	−32	92	8.7	9.5	37	−0.6	−2	117	2.4	2.0	31	1.4	4.6
NorESM1-M	−0.7	−0.1	16	19.6	−4.7	−24	91	7.8	8.5	39	2.0	5	117	3.8	3.3	32	1.9	6.0
**MM Mean**	**0.2**	**−0.6**	**−366**	**6.8**	**−3.4**	**−50**	**101**	**2.9**	**2.9**	**37**	**−4.5**	**−12**	**119**	**−2.9**	**−2.5**	**33**	**−1.8**	**−5.6**
**MM Median**	**0.2**	**−0.6**	**−238**	**5.6**	**−3.3**	**−58**	**105**	**4.3**	**4.1**	**37**	**−3.6**	**−10**	**128**	**−1.7**	**−1.3**	**35**	**−1.5**	**−4.3**

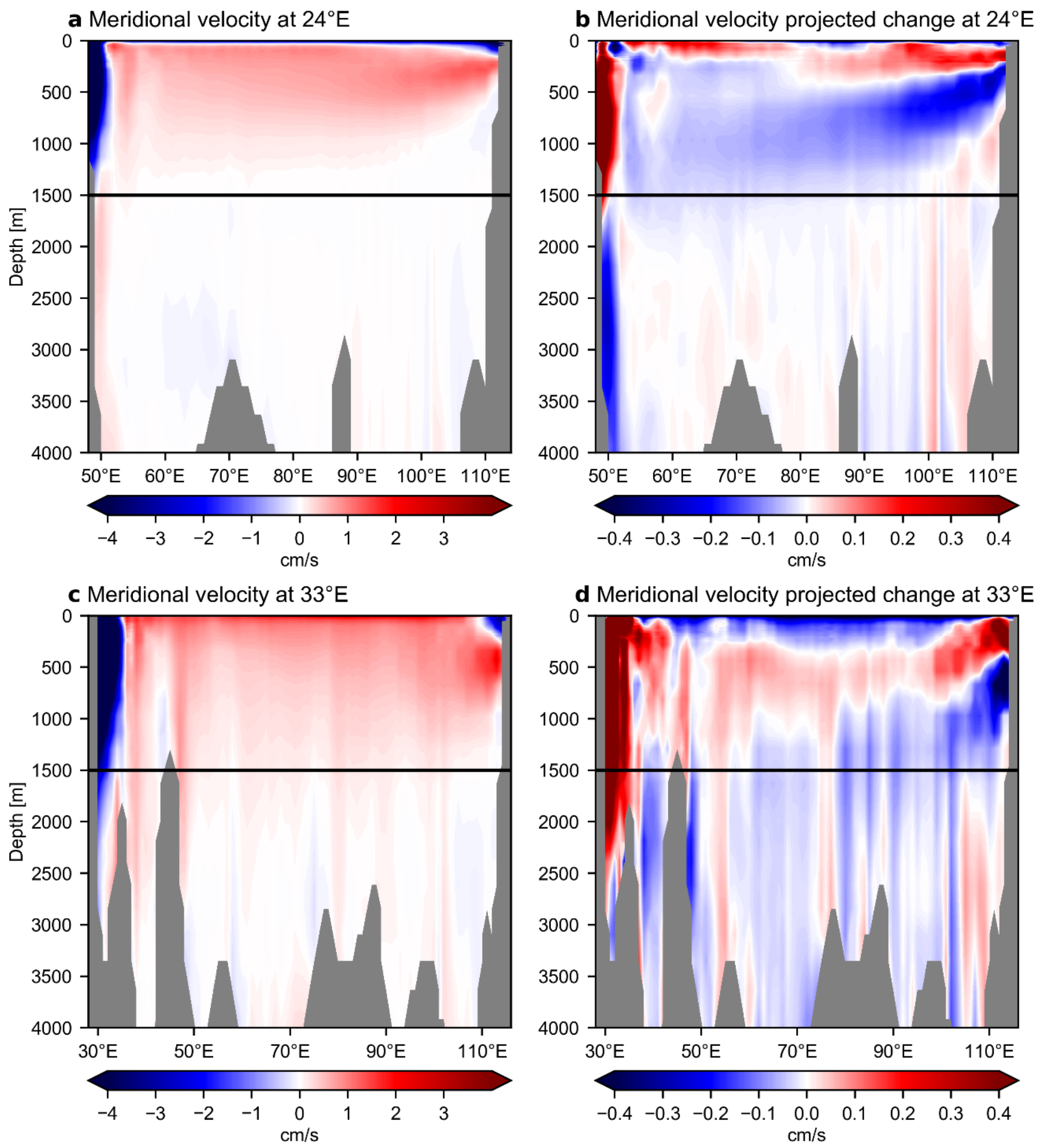

Figure S1. South Indian Ocean multi-model mean meridional velocity. (**a**) Annual mean velocity (cm/s) at 24°S between Madagascar and Australia in the historical scenario. (**b**) Same as (**a**) but for the projected change (RCP8.5 minus historical). (**c**) Annual mean velocity at 33°S between Africa and Australia in the historical scenario. (**d**) Same as (**c**), but for the projected change. (**a,c**) Negative values (blue) indicate southward velocity and a corresponding positive change (red) in (**b,d**) indicates a decrease in velocity. Note the multi-model mean projected change patterns in (**c,d**) are similar for individual models.

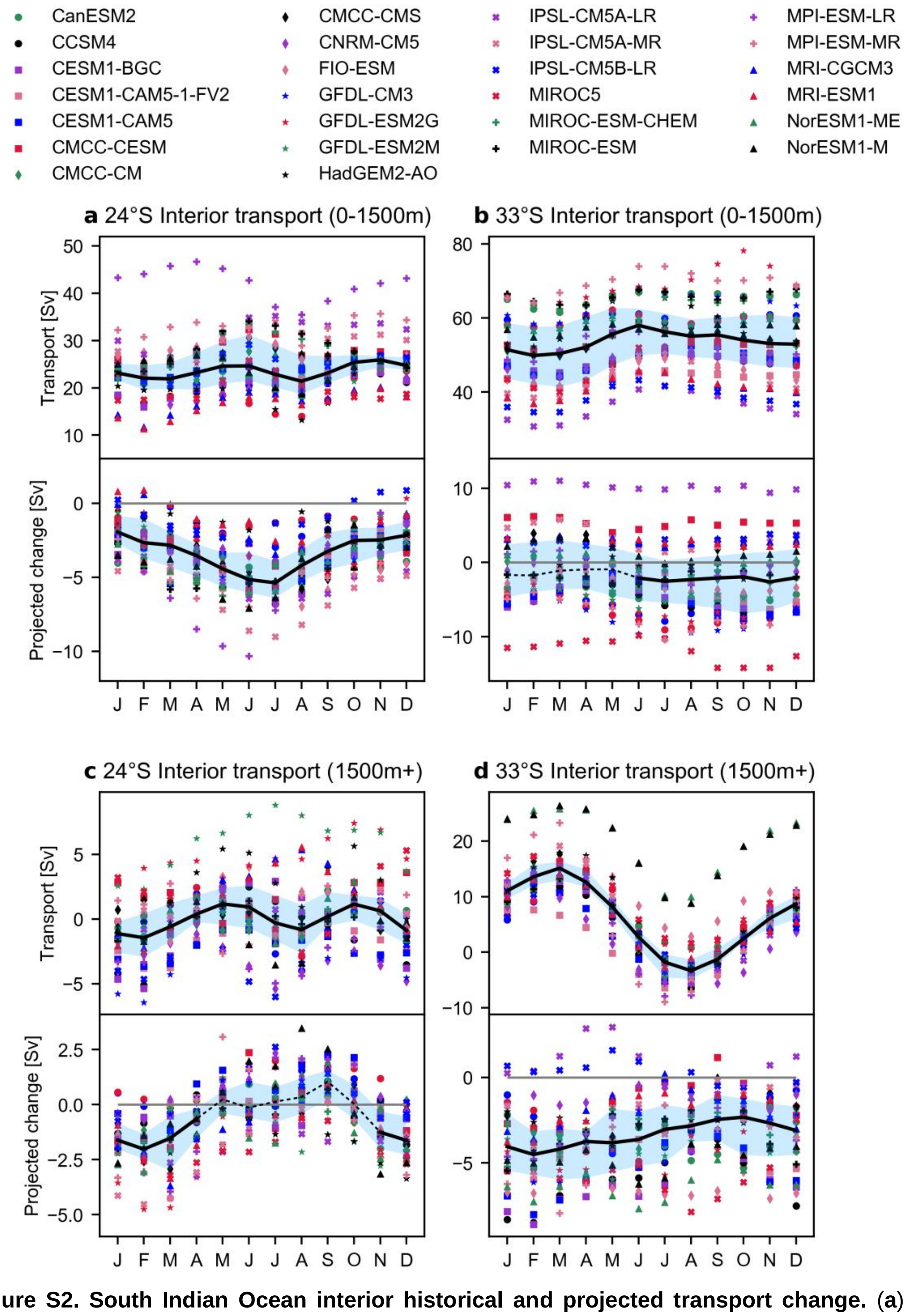

Figure S2. South Indian Ocean interior historical and projected transport change. (**a**) Upper-interior monthly transport (Sv) at 24°S in the historical scenario (top panel) and projected change (bottom panel). (**b**) Same as (**a**), but at 33°S. (**c**) Same as (**a**), but for deep interior transport. (**d**) Same as (**c**), but at 33°S. Shown are the multi-model median (black line), interquartile range (shaded blue), individual model (markers; see legend at top) transport estimates and, in the bottom panels, a grey zero-line for reference. Northward historical transport is represented by positive values. Projected changes that are not statistically significant at the 95% level are indicated by dashed lines.

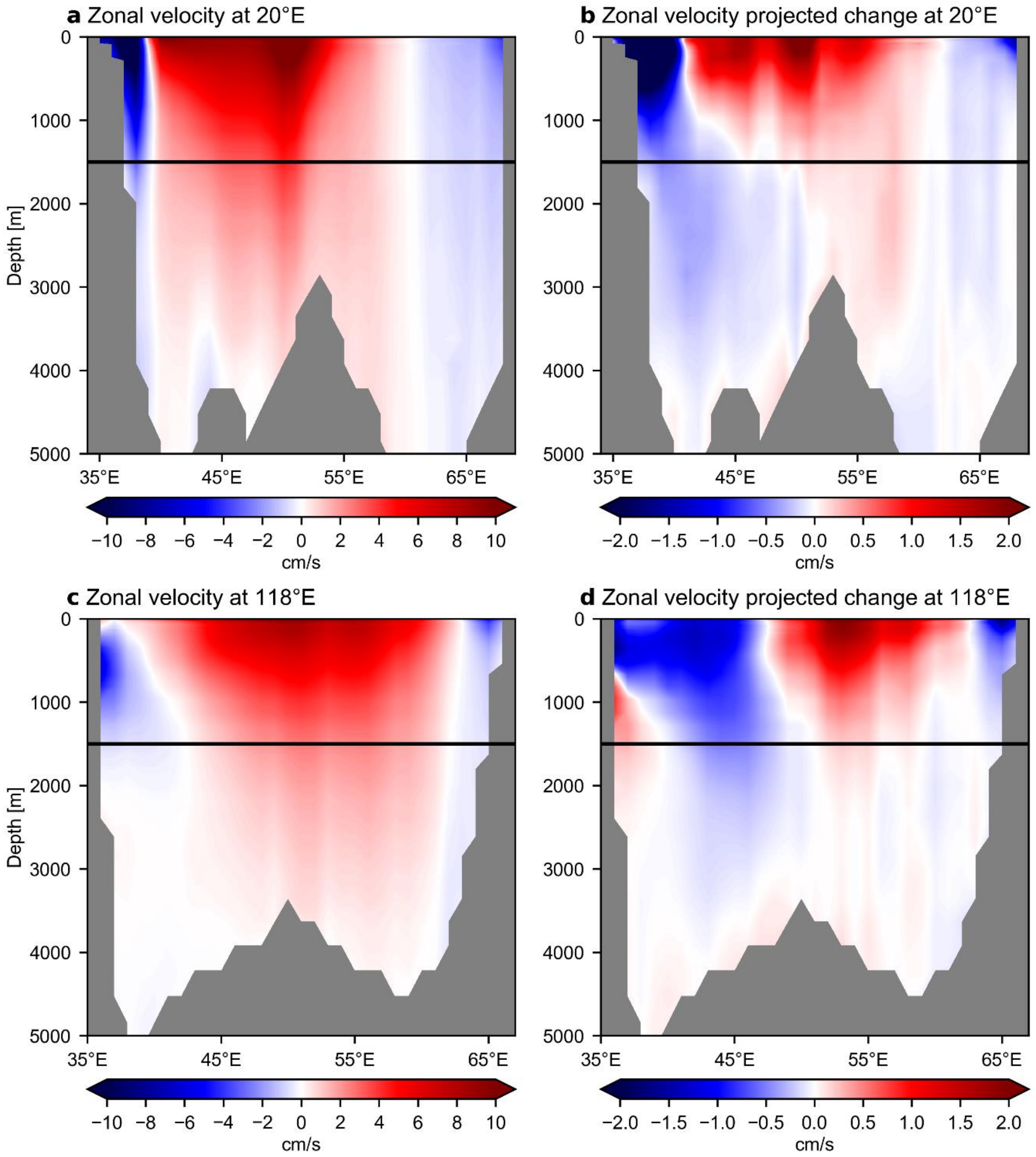

Figure S3. Southern Ocean multi-model mean zonal velocity. (**a**) Annual mean velocity (cm/s) at 20°E between Africa and Antarctica in the historical scenario. (**b**) Same as (**a**), but for the projected change (RCP8.5 minus historical). (**c**) Annual mean velocity at 118°E between Australia and Antarctica in the historical scenario. (**d**) Same as (**c**), but for the projected change. (**a,c**) Negative values (blue) indicates westward velocity and a corresponding positive change (red) in (**b,d**) indicates a decrease in velocity. Note the multi-model mean projected change patterns in (**c,d**) are similar for individual models.

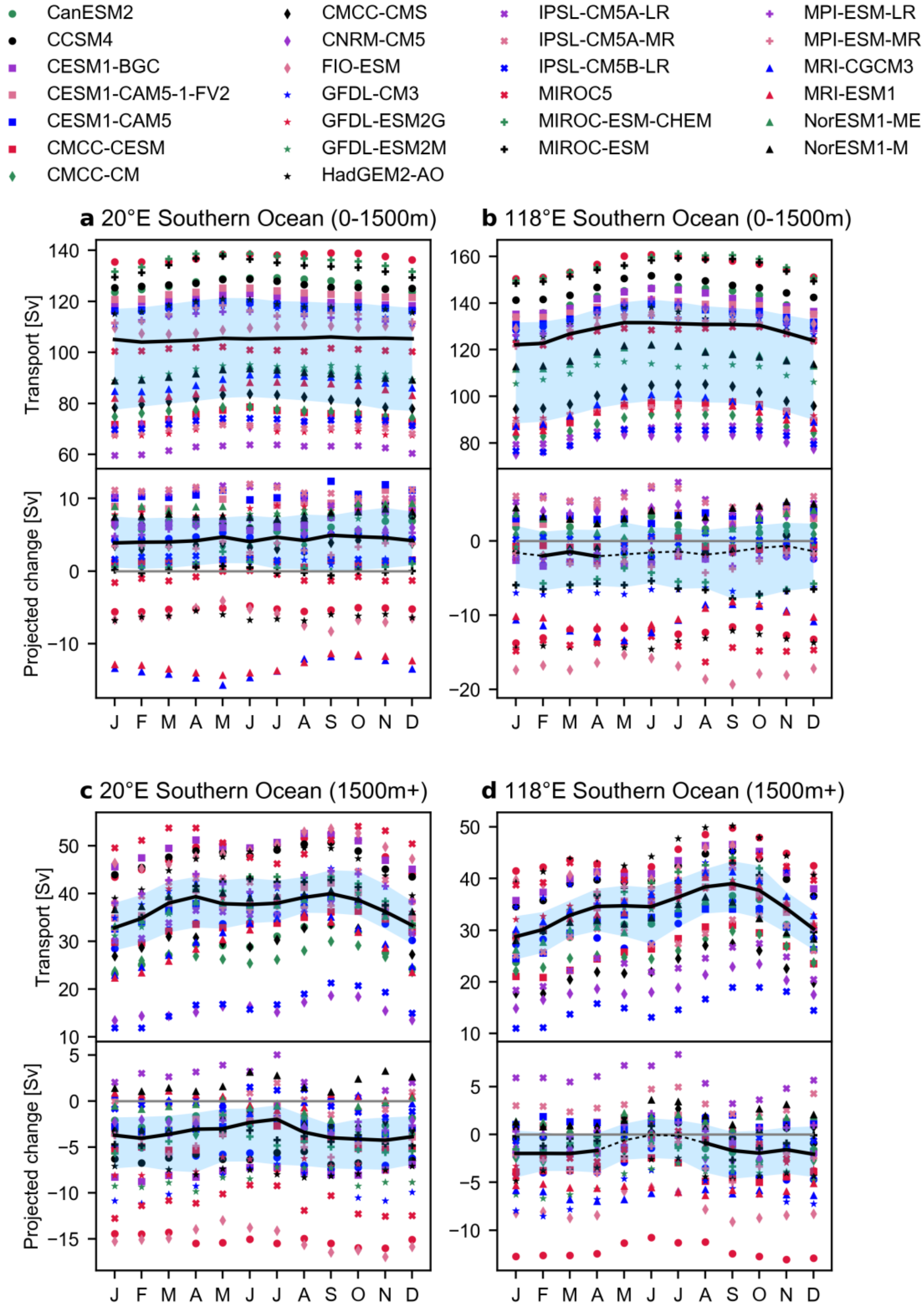

Figure S4. Southern Ocean historical and projected transport change. (**a**) Upper-interior monthly transport (Sv) at 20°E in the historical scenario (top panel) and projected change (bottom panel). (**b**) Same as (**a**), but at 118°E. (**c**) Same as (**a**), but for deep interior transport. (**d**) Same as (**c**), but at 118°E. Shown are the multi-model median (black line), interquartile range (shaded blue), individual model (markers; see legend at top) transport estimates and, in the bottom panels, a grey zero-line for reference. Eastward transport in the historical scenario is represented by positive values. Projected changes that are not statistically significant at the 95% level are indicated by dashed lines.

References

1. Bi, D. *et al.* The ACCESS Coupled Model: Description, Control Climate and Evaluation. *Aust. Meteorol. Oceanogr. J.* **63**, 41–64 (2013).
2. Arora, V. K. *et al.* Carbon emission limits required to satisfy future representative concentration pathways of greenhouse gases. *Geophys. Res. Lett.* **38**, (2011).
3. Gent, P. R. *et al.* The Community Climate System Model Version 4. *J. Clim.* **24**, 4973–4991 (2011).
4. Long, M. C., Lindsay, K., Peacock, S., Moore, J. K. & Doney, S. C. Twentieth-Century Oceanic Carbon Uptake and Storage in CESM1(BGC)*. *J. Clim.* **26**, 6775–6800 (2013).
5. Hurrell, J. W. *et al.* The Community Earth System Model: A Framework for Collaborative Research. *Bull. Am. Meteorol. Soc.* **94**, 1339–1360 (2013).
6. Fogli, P. G. *et al. INGV-CMCC Carbon (ICC): A Carbon Cycle Earth System Model*. *CMCC Research Paper 61* (2009). doi:10.2139/ssrn.1517282
7. Scoccimarro, E. *et al.* Effects of Tropical Cyclones on Ocean Heat Transport in a High-Resolution Coupled General Circulation Model. *J. Clim.* **24**, 4368–4384 (2011).
8. Voldoire, A. *et al.* The CNRM-CM5.1 global climate model: description and basic evaluation. *Clim. Dyn.* **40**, 2091–2121 (2013).
9. Huang, C. J., Qiao, F., Shu, Q. & Song, Z. Evaluating austral summer mixed-layer response to surface wave-induced mixing in the Southern Ocean. *J. Geophys. Res. Ocean.* **117**, C00J18 (2012).
10. Donner, L. J. *et al.* The Dynamical Core, Physical Parameterizations, and Basic Simulation Characteristics of the Atmospheric Component AM3 of the GFDL Global Coupled Model CM3. *J. Clim.* **24**, 3484–3519 (2011).
11. Martin, G. M. *et al.* The HadGEM2 family of Met Office Unified Model climate configurations. *Geosci. Model Dev.* **4**, 723–757 (2011).
12. Dufresne, J. L. *et al. Climate change projections using the IPSL-CM5 Earth System Model: From CMIP3 to CMIP5*. *Clim. Dyn.* **40**, (2013).
13. Watanabe, M. *et al.* Improved Climate Simulation by MIROC5: Mean States, Variability, and Climate Sensitivity. *J. Clim.* **23**, 6312–6335 (2010).
14. Watanabe, S. *et al.* MIROC-ESM 2010: Model description and basic results of CMIP5-20c3m experiments. *Geosci. Model Dev.* **4**, 845–872 (2011).
15. Raddatz, T. J. *et al.* Will the tropical land biosphere dominate the climate-carbon cycle feedback during the twenty-first century? *Clim. Dyn.* **29**, 565–574 (2007).
16. Yukimoto, S. *et al.* A New Global Climate Model of the Meteorological Research Institute: MRI-CGCM3 -Model Description and Basic Performance-. *J. Meteorol. Soc. Japan* **90A**, 23–64 (2012).
17. Iversen, T. *et al.* The Norwegian Earth System Model, NorESM1-M – Part 2: Climate response and scenario projections. *Geosci. Model Dev.* **5**, 2933–2998 (2012).
18. Godfrey, J. S. & Golding, T. J. The Sverdrup Relation in the Indian Ocean, and the Effect of Pacific-Indian Ocean Throughflow on Indian Ocean Circulation and on the East Australian

Current. *J. Phys. Oceanogr.* **11**, 771–779 (1981).

19. Godfrey, J. S. A Sverdrup model of the depth-integrated flow for the world ocean allowing for island circulations. *Geophys. Astrophys. Fluid Dyn.* **45**, 89–112 (1989).
20. Ganachaud, A. & Wunsch, C. Improved estimates of global ocean circulation, heat transport and mixing from hydrographic data. *Nature* **408**, 453–456 (2000).
21. Sprintall, J., Wijffels, S. E., Molcard, R. & Jaya, I. Direct estimates of the Indonesian Throughflow entering the Indian Ocean: 2004–2006. *J. Geophys. Res.* **114**, C07001 (2009).
22. Gordon, A. L. *et al.* The Indonesian throughflow during 2004-2006 as observed by the INSTANT program. *Dyn. Atmos. Ocean.* **50**, 115–128 (2010).
23. Thompson, R. O. R. Y. Observations of the Leeuwin Current off Western Australia. *J. Phys. Oceanogr.* **14**, 623–628 (1984).
24. Furue, R., Guerreiro, K., Phillips, H. E., McCreary, J. P. & Bindoff, N. L. On the Leeuwin Current System and Its Linkage to Zonal Flows in the South Indian Ocean as Inferred from a Gridded Hydrography. *J. Phys. Oceanogr.* **47**, 583–602 (2017).
25. Smith, R. L., Huyer, A., Godfrey, J. S. & Church, J. A. The Leeuwin Current off Western Australia, 1986–1987. *J. Phys. Oceanogr.* **21**, 323–345 (2002).
26. Feng, M., Meyers, G., Pearce, A. & Wijffels, S. Annual and interannual variations of the Leeuwin Current at 32°S. *J. Geophys. Res.* **108**, 3355 (2003).
27. Swallow, J., Fieux, M. & Schott, F. The Boundary Currents East and North of Madagascar 1. Geostrophic Currents and Transports. *J. Geophys. Res.* **93**, 4951–4962 (1988).
28. Schott, F. A., Fieux, M., Kindle, J., Swallow, J. & Zantopp, R. The Boundary Currents East and North of Madagascar 2. Direct Measurements and Model Comparisons. *J. Geophys. Res.* **93**, 4963–4974 (1988).
29. Voldsund, A., Aguiar-González, B., Gammelsrød, T., Krakstad, J.-O. & Ullgren, J. Observations of the East Madagascar Current system: Dynamics and volume transports. *J. Mar. Res.* **75**, 531–555 (2017).
30. Ponsoni, L., Aguiar-González, B., Ridderinkhof, H. & Maas, L. R. M. The East Madagascar Current: Volume Transport and Variability Based on Long-Term Observations. *J. Phys. Oceanogr.* **46**, 1045–1065 (2016).
31. Donohue, K. A. & Toole, J. M. A near-synoptic survey of the Southwest Indian Ocean. *Deep-Sea. Res. Pt. II* **50**, 1893–1931 (2003).
32. Nauw, J. J., van Aken, H. M., Webb, A., Lutjeharms, J. R. E. & de Ruijter, W. P. M. Observations of the southern East Madagascar Current and undercurrent and countercurrent system. *J. Geophys. Res.* **113**, C08006 (2008).
33. de Ruijter, W. P. M., Ridderinkhof, H., Lutjeharms, J. R. E., Schouten, M. W. & Veth, C. Observations of the flow in the Mozambique Channel. *Geophys. Res. Lett.* **29**, 1502 (2002).
34. Ridderinkhof, H. *et al.* Seasonal and interannual variability in the Mozambique Channel from moored current observations. *J. Geophys. Res.* **115**, C06010 (2010).
35. DiMarco, S. F. *et al.* Volume transport and property distributions of the Mozambique Channel. *Deep-Sea. Res. Pt. II* **49**, 1481–1511 (2002).

36. Bryden, H. L., Beal, L. M. & Duncan, L. M. Structure and Transport of the Agulhas Current and Its Temporal Variability. *J. Oceanogr.* **61**, 479–492 (2005).
37. Sloyan, B. & Rintoul, S. R. Circulation, Renewal, and Modification of Antarctic Mode and Intermediate Water. *J. Phys. Oceanogr.* **31**, 1005–1030 (2001).
38. Toole, J. M. & Warren, B. A. A hydrographic section across the subtropical South Indian Ocean. *Deep-Sea. Res. Pt. I* **40**, 1973–2019 (1993).
39. Macdonald, A. M. The global ocean circulation: a hydrographic estimate and regional analysis. *Prog. Oceanogr.* **41**, 281–382 (1998).
40. Ganachaud, A., Wunsch, C., Marotzke, J. & Toole, J. Meridional overturning and large-scale circulation of the Indian Ocean. *J. Geophys. Res. Ocean.* **105**, 26117–26134 (2000).
41. Robbins, P. E. & Toole, J. M. The dissolved silica budget as a constraint on the meridional overturning circulation of the Indian Ocean. *Deep-Sea Res. Pt. I* **44**, 879–906 (1997).
42. Beal, L. M., Elipot, S., Houk, A. & Leber, G. M. Capturing the Transport Variability of a Western Boundary Jet: Results from the Agulhas Current Time-Series Experiment (ACT). *J. Phys. Oceanogr.* **45**, 1302–1324 (2015).
43. Krug, M. & Tournadre, J. Satellite observations of an annual cycle in the Agulhas Current. *Geophys. Res. Lett.* **39**, L15607 (2012).
